# Distribution and function of prokaryotes involved in mercury methylation, demethylation, and reduction in the western North Pacific Subtropical Gyre

**DOI:** 10.3389/fmicb.2025.1642479

**Published:** 2026-01-22

**Authors:** Yuya Tada, Ryota Nakajima, Minoru Kitamura, Kohji Marumoto

**Affiliations:** 1 Department of Environment and Public Health, National Institute for Minamata Disease (NIMD), Kumamoto, Japan; 2 Research Institute for Global Change, Japan Agency for Marine-Earth Science and Technology (JAMSTEC), Yokosuka, Japan

**Keywords:** *hgcAB*, marine bacteria, *mer*A, *merB*, mercury, methylmercury

## Abstract

Methylmercury (MeHg), a bioaccumulative neurotoxic heavy metal, substantially threatens environmental and human health. In natural environments, MeHg formation and degradation are primarily mediated by microorganisms containing *hgcAB*, *merA*, or *merB* genes. However, these genes have not been simultaneously analyzed in open-ocean samples. This study aimed to investigate the distribution and phylogeny of functional genes associated with mercury (Hg) methylation (*hgcA* and *hgcB*), demethylation (*merB*), and reduction (*merA*), as well as dissolved total Hg (THg) and MeHg concentrations in the western North Pacific Subtropical Gyre (WNPSG) using metagenomic analysis. Although THg levels varied across sampling sites, MeHg concentrations consistently increased with depth. A strong correlation between dissolved MeHg and apparent oxygen utilization indicated a link between Hg methylation and microbial respiration. *hgcA*, *merB*, and *merA* were predominantly detected at depths of 500–1,500 m, where MeHg concentrations peaked, indicating active microbial Hg speciation within mesopelagic layers. A higher abundance of *hgcA* than *merB* suggests that microbial Hg methylation may surpass demethylation in this region. Phylogenetic analyses of *hgcAB* identified the *Nitrospina* lineage as dominant Hg methylators. Metabolic pathway analyses of metagenome-assembled genomes (MAGs) showed that *Nitrospina* harboring *hgcAB* possesses the nitrite reductase pathway, suggesting a linkage between Hg methylation and nitrogen cycling. MAGs with *hgcA* affiliated with *Myxococcota* (Deltaproteobacteria) exhibited a strong association with sulfur cycling. Diverse lineages harboring *merB* and *merA* genes were identified, suggesting that MeHg demethylation and Hg(II) reduction likely co-occur. Methanogenesis pathways in some Alphaproteobacteria with *merB* or *merA* suggest a potential connection between methane production and MeHg degradation and Hg(II) reduction. These findings provide novel insights into the intricate interactions between microbial communities, functional gene distributions, and Hg biogeochemical cycling in the WNPSG.

## 1 Introduction

Mercury (Hg) is a globally concerning toxic metal, primarily released through fossil fuel combustion, artisanal gold mining, and cement production (Outridge et al., 2018). Atmospheric Hg is transported long distances and deposited via precipitation into terrestrial and marine environments, eventually accumulating in the oceans, where Hg is transformed into methylmercury (MeHg), a highly neurotoxic compound, through biological and abiotic processes. MeHg gradually bioaccumulates in marine organisms such as plankton, fish, and marine mammals via the marine food web, thereby posing serious risks to humans. As fluctuations in Hg concentrations in fish and shellfish depend on bioaccumulating MeHg concentrations rather than non-bioaccumulating inorganic Hg (Mason et al., 1995), information on MeHg production and loss in the marine environment is essential for understanding the Hg cycle in marine ecosystems.

Previous oceanic surveys have confirmed a depth gradient in seawater MeHg concentrations, with consistently higher MeHg concentrations in the mid-depth layers (approximately 200–1,000 m depth) in the northern Pacific Ocean (Sunderland et al., 2004; Munson et al., 2015; Kim et al., 2017). This gradient has also been observed in the Subtropical Gyre of the North Pacific (Hammerschmidt and Bowman, 2012), one of the largest oligotrophic areas in the world ocean. According to Bowman et al. (2020), factors contributing to the formation of such MeHg concentration gradients include vertical and horizontal transport and microbial activities such as organic matter decomposition and oxygen consumption. Especially, a positive correlation between MeHg concentrations and apparent oxygen utilization (AOU) has been observed in the open-ocean environments, suggesting that MeHg may be produced by microbial remineralization of organic matter (Mason and Fitzgerald, 1993; Sunderland et al., 2004; Hammerschmidt and Bowman, 2012; Kim et al., 2017; Bowman et al., 2020). However, the identity and ecological roles of MeHg-producing microbes in the North Pacific Subtropical Gyre remain unknown.

**Figure 1:**
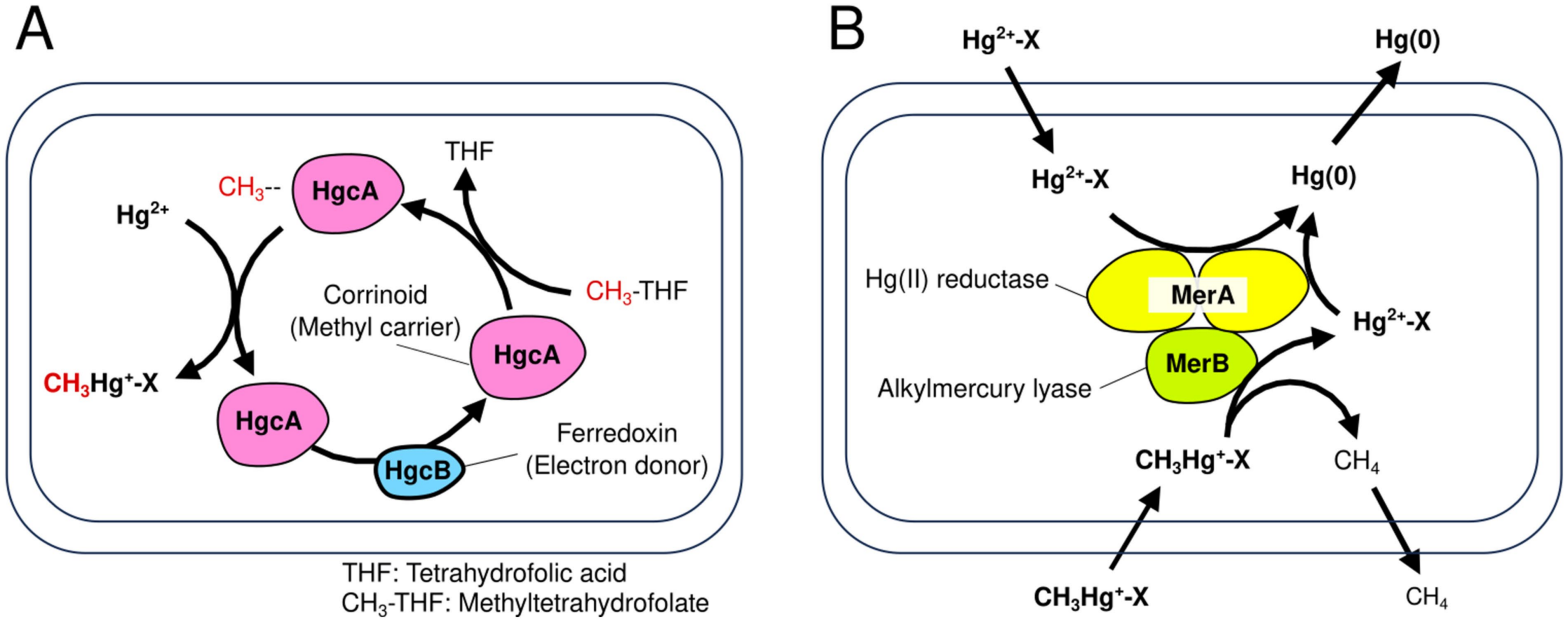
Schematic of microbial mercury (Hg) transformations showing Hg methylation **(A)** and MeHg demethylation coupled to Hg(II) reduction **(B)**. In **(A)**, the CH_3_ group is transferred from methyltetrahydrofolate to Hg(II) by HgcA (corrinoid protein), while HgcB (ferredoxin protein) donates electrons to regenerate the active form of HgcA; in **(B)**, MerB (organomercury lyase) catalyzes protonolysis of the Hg–carbon bond in organomercury compounds to yield Hg(II), which is subsequently reduced to Hg(0) by MerA [Hg(II) reductase]. Figures were created based on Parks et al. (2013), and Boyd and Barkay, (2012).

Microbial Hg methylation involves a gene pair: *hgcA*, which encodes a corrinoid protein that serves as the methyl carrier, and *hgcB*, which encodes a ferredoxin protein that serves as the electron donor (Parks et al., 2013) (Figure 1A). In addition to previously validated cultured strains—primarily anaerobic microorganisms—with Hg methylation capacity (Compeau and Bartha, 1985; Gilmour and Henry, 1991; Kerry et al., 1991; Hamelin et al., 2011; Gilmour et al., 2013), recent genome analyses—including metagenome-assembled genome (MAGs), which were reconstructed from environmental samples—have identified several uncultured lineages such as Planctomycetota, Verrucomicrobiota, Chloroflexota, Nitrospinota, and certain archaea, indicating that diverse prokaryotes may possess the potential for Hg methylation (Podar et al., 2015; Gionfriddo et al., 2019; Bravo and Cosio, 2020). MeHg demethylation and Hg(II) reduction are associated with *merB* and *merA*, encoding alkylmercury lyase and Hg(II) reductase, respectively (Boyd and Barkay, 2012) (Figure 1B). Similar to *hgcAB*, *merA* and *merB* have been identified in various bacterial and archaeal genomes (Christakis et al., 2021). Quantitative analysis of these genes is crucial to advancing the understanding of microbial roles in Hg transformation in environmental systems.

The *Nitrospina*, *Desulfobacterales*, *Chloroflexi*, *Firmicutes*, *Spirochaetes*, *Marinimicrobia*, *Verrucomicrobia*, *Calditrichaeota*, *Kiritimatiellaeota* lineages with *hgcAB* genes contribute to Hg methylation in marine environments (Villar et al., 2020; Capo et al., 2020; Tada et al., 2020; Tada et al., 2021; Lin et al., 2021). Furthermore, Actinomycetes, Alphaproteobacteria, Gammaproteobacteria, and Bacteroidota with *merA* or *merB* could be key players in MeHg demethylation and Hg(II) reduction (Sanz-Sáez et al., 2022). However, simultaneous analysis of these genes within the same open ocean samples remains limited, thereby constraining a comprehensive understanding of their ecological roles. Therefore, integrated metagenomic approaches are essential to assess the full microbial contribution to Hg methylation, demethylation, and reduction. Furthermore, functional gene analysis within MAGs harboring Hg-related genes can reveal linkages between microbial mercury speciation and broader biogeochemical processes in the ocean.

This study aimed to investigate Hg transformations in the mesopelagic layers of the western North Pacific Subtropical Gyre (WNPSG) by measuring dissolved THg and MeHg (dTHg and dMeHg, respectively) concentrations, analyzing *hgcAB*, *merA*, and *merB* distribution via metagenomics, and characterizing Hg-related microbial pathways using MAG-based functional analysis.

## 2 Materials and methods

### 2.1 Research cruise and seawater sampling

Seawater samples were collected from three stations (Sts. 1, 6, and 9) in the western North Pacific (Supplementary Figure S1) between November 3 and 27, 2021, at depths ranging from 200 to 1,500 m (Table 1). Seawater was collected using a Niskin-X sampler with a Teflon-coated inner wall and Kevlar line installed on the ship. Samples were filtered through an AcroPack in-line filter (0.22 μm pore size) to remove particulates. Filtered seawater was stored in 0.1 L and 0.5 L PFA bottles for THg and MeHg analysis, respectively. For THg analysis, 1.0 mL of concentrated ultrapure HCl (Kanto Chemical Co., Tokyo, Japan) and BrCl (Guaranteed Reagent, Kanto Chemical Co.) were added to achieve a final concentration of approximately 0.002 mol L^−1^ (Marumoto and Imai, 2015). For MeHg analysis, 2.0 mL of 10 M reagent-grade H₂SO₄ (Kanto Chemical Co.) was added (Marumoto et al., 2018). Treated samples were stored at 4 °C in the dark.

**Table 1:** Sampling position in the western North Pacific Subtropical Gyre.

Station	Date	Position	Sampling depth (m)	Maximum depth (m)
1	07/11/2021	36°00’N, 160°00′E	200, 500, 1,000, 1,500	4,695
6	11/11/2021	25°00’N, 160°00′E	200, 500, 967, 1,500	5,808
9	14/11/2021	30°00’N, 165°00′E	200, 500, 1,175, 1,500	5,910

Seawater samples for counting prokaryotic cells were preserved with 0.5% glutaraldehyde in 15 mL polypropylene tubes and stored at −80 °C. For metagenomic analysis, 5 L seawater samples were filtered through a 0.22-μm-pore-size Sterivex cartridge filter (Millipore, Burlington, MA, United States) to capture prokaryotic cells. The filters were stored at −80 °C.

Macronutrient samples were collected in acrylic tubes and stored at −30 °C until analysis. Nitrate plus nitrite, phosphate, and silicic acid concentrations were measured using a QuAAtro39 segmented continuous flow analyzer (Bran + Luebbe, Norderstedt, Germany).

Seawater physicochemical parameters, including temperature, salinity, chlorophyll a, and dissolved oxygen concentrations, were recorded using a conductivity, temperature, and depth (CTD) system (SBE9plus CTD system, Sea-Bird Electronics). The depth of the subsurface chlorophyll maximum layer was determined from the CTD profiles.

### 2.2 dTHg and dMeHg analyses

The dTHg and dMeHg analyses were performed as previously described (Marumoto et al., 2018). dTHg was quantified using EPA Method 1,631 (U.S. Environmental Protection Agency, 2002) via cold vapor atomic fluorescence spectrometry with gold amalgamation (RA-FG+; Nippon Instruments Corporation) after Hg(0) generation with 1 mL of 20% (w/v) SnCl₂ as a reducing agent. Analytical precision was validated through multiple measurements of BCR579 standard reference material (certified range: 1900 ± 500 pg. L^−1^; 9.5 ± 2.5 pM), yielding values of 1850 ± 60 pg. L^−1^ (9.3 ± 0.3 pM, n = 6), consistently within the certified range. The method detection limit, calculated from ultrapure water blanks and defined as three times the standard deviation of the blanks, was 7.03 pg. L^−1^ (0.035 pM, *n* = 11). The mean blank dTHg concentration was 16 ± 2.3 pg. L^−1^ (0.08 ± 0.01 pM, *n* = 11).

The dMeHg determination was performed using solvent extraction with dithizone–toluene and Na₂S solutions (Ministry of the Environment, Japan, Mercury Analysis Manual, 2004). dMeHg concentrations in Na₂S solutions were measured via ethylation with NaB(C₂H₅)₄, preconcentration onto a Tenax trap, thermal desorption, and gas chromatography with atomic fluorescence detection as previously described (Logar et al., 2002). The method detection limit for dMeHg, calculated as for dTHg, was 1.4 pg. L^−1^ (0.007 pM), with ultrapure water blanks yielding 0.85 ± 0.46 pg. L^−1^ (0.004 ± 0.002 pM, *n* = 3). MeHg recovery of dithizone–toluene was 99% ± 3% (*n* = 7), validated by spiking experiments using a 1 ng mL^−1^ alkaline-dissolved solution obtained from DORM-2 (certified range: 4.47 ± 0.32 mg kg^−1^ dry weight), an international reference material for MeHg in dogfish. One DORM-2 solution was analyzed for every seven samples. At St. 1 (1,500 m), St. 6 (967 m), and St. 9 (1,175 m), duplicate samples were collected, and the analytical precision of these duplicates was 9.4, 10.4, and 4.7%, respectively.

### 2.3 Prokaryotic cell abundance

The prokaryotic cells were stained with 4′,6-diamidino-2-phenylindole and filtered with a 0.22 μm pore-size polycarbonate filter (GTTP00250, Millipore). Cells were counted under an epifluorescence microscope (10 fields per sample).

### 2.4 DNA extraction

Environmental DNA was extracted from the Sterivex cartridge filter using a PowerWater DNA Isolation Kit (Qiagen, Hilden, Germany). Before extraction, the Sterivex cartridge was opened, and the filter was cut into 16 pieces, which were transferred to PowerWater bead tubes for DNA extraction following the manufacturer’s protocol. DNA solutions were treated with RNase A (final concentration: 0.1 μg μL^−1^; Promega, Madison, WI, United States). The extracted DNA was used for 16S rRNA deep sequencing and shotgun metagenomic sequencing and was stored at −80 °C. The quality of metagenomic DNA was assessed via 1% agarose gel electrophoresis.

### 2.5 16S rRNA gene deep-sequencing analysis

Bacterial and archaeal 16S rRNA (V4 region) gene fragments were amplified using the following primers with adaptor sequences from Illumina (San Diego, CA, USA): 515F, ACACTCTTTCCCTACACGACGCTCTTCCGATCT-GTGCCAGCMGCCGCGGTAA; and 806RB, GTGACTGGAGTTCAGACGTGTGCTCTTCCGATCT-GGACTACNVGGGTWTCTAAT (Caporaso et al., 2011; Apprill et al., 2015). The PCR program included an initial denaturation step for 5 min at 94 °C, followed by 25 cycles of denaturation (94 °C, 30 s), annealing (50 °C, 30 s), and extension (72 °C, 30 s); a final extension for 3 min at 72 °C completed the amplification reaction. The amplicons were visualized using electrophoresis on SYBR Gold-stained 1.5% agarose gels. The PCR amplicons were then sequenced considering 2 × 250-bp paired-end sequences on the Illumina MiSeq platform. The raw 16S rRNA sequence data have been deposited in the DNA Data Bank of Japan-Sequence Read Archive (DDBJ-SRA) under the accession number (DRR683897-DRR683908).

Quality filtering for noise and short read sequences removal was completed in the QIIME pipeline.^1^ Chimeras were identified and removed with USEARCH (Edgar et al., 2011) using the Greengenes16S rRNA gene dataset (McDonald et al., 2012) as a reference. High-quality sequences were clustered into operational taxonomic units at a 97% similarity threshold.

### 2.6 Metagenome sequencing

Metagenomic DNA was barcoded per sample and used for library preparation. Paired-end libraries (~350 bp insert size) were constructed using the TruSeq Nano DNA Library Prep Kit (Illumina) following the manufacturer’s protocol. Sequencing was conducted by Macrogen Japan Corporation (Tokyo, Japan) on the Illumina NovaSeq 6,000 platform using 2 × 250-bp paired-end sequencing. Raw metagenomic sequence data have been deposited in the DDBJ-SRA (accession numbers: DRR683870-DRR683881).

### 2.7 Metagenomic sequence data analyses

The detailed metagenomic sequence analysis of Hg-related genes is presented in Tada et al. (2023). Raw metagenomic sequences were processed using the fastq preprocessor “fastp” (Chen et al., 2018) to remove Illumina adapters, low-quality sequences (*Q*-value > 30; sequence length > 25 bp), and polyG tails. After quality filtering, contigs were assembled using MEGAHIT (Li et al., 2016) with default parameters. Protein-coding genes were identified from the metagenomic contigs using Prodigal 2.6.3 (Hyatt et al., 2010). To detect *hgcA* and *hgcB* sequences, a Hidden Markov Model (HMM) profile was generated using HMMER v3.2.1 (Eddy, 2009; Finn et al., 2011) with e-value thresholds of 10^−5^ for *hgcAB* and *merB* and 10^−10^ for *merA*. The HMM profile was constructed using representative sequences for *hgcAB* (Gionfriddo et al., 2019), *merA*, and *merB* (Christakis et al., 2021). The specificity of the HMMs for Hg-related gene sequences was validated through a local search using hmmsearch (HMMER v3.2.1) with reference sequences. Sequences lacking conserved functional motifs were excluded: *hgcA*-like sequences without the conserved cysteine C93 motif (Gionfriddo et al., 2016; Parks et al., 2013; Smith et al., 2015), *hgcB* lacking two strictly conserved CX_2_CX_2_CX_3_C motifs (Gionfriddo et al., 2016; Smith et al., 2015), *merA* without a conserved cysteine pair at positions 207 and 212 (CX_4_C motif), tyrosine at position 264, tyrosine or phenylalanine (for Bacteria and Archaeota, respectively) at position 605, and vicinal cysteine pair at position 628 and 629 in *merA* from *Bacillus* sp. RC607 (Boyd and Barkay, 2012; Christakis et al., 2021) (Supplementary Figure S2A), and *merB* without Cys-96, Asp-99, Cys-159, and Cys-117 (Christakis et al., 2021; Lafrance-Vanasse et al., 2009; Pitts and Summers, 2002) (Supplementary Figure S2B). *hgcAB*, *merA*, and *merB* abundances in metagenomic sequences were normalized to the abundance of *rpoB* (TIGR02013), a conserved single-copy bacterial gene.

After redundant sequences were removed with CD-HIT (Li and Godzik, 2006) at a 90% identity cutoff, amino acid sequences were aligned using MAFFT with the UPGMA clustering method for phylogenetic analysis (Katoh and Standley, 2013). Phylogenetic trees were constructed using FastTree (Price et al., 2009) and visualized with iTOL (Letunic and Bork, 2019).

MAGs were constructed using MetaBat2 (Kang et al., 2019) and SemiBin2 (Pan et al., 2023) with default parameters and refined with Binning Refiner (Song and Thomas, 2017). MAG completeness and contamination scores were assessed using CheckM v1.0.7–13 (Parks et al., 2015). Taxonomic classification of MAGs was assigned using GTDB-tk v2 (Chaumeil et al., 2022). Metabolic pathways of MAGs with Hg-related genes were predicted using GhostKOALA.^2^ The completeness of each pathway was estimated and visualized using KEGG-decoder.^3^

### 2.8 Statistical analyses

Data were analyzed with R software (v.3.4.3; Pinheiro et al., 2017) using Spearman’s rank correlation analysis with the function *cor.test()* in the stats package. A heatmap of functional pathways of MAGs was constructed using the ggplot2 (Villanueva and Chen, 2019) in R after standardization.

## 3 Results

### 3.1 Vertical profiles of environmental characteristics, dTHg, and dMeHg

**Figure 2:**
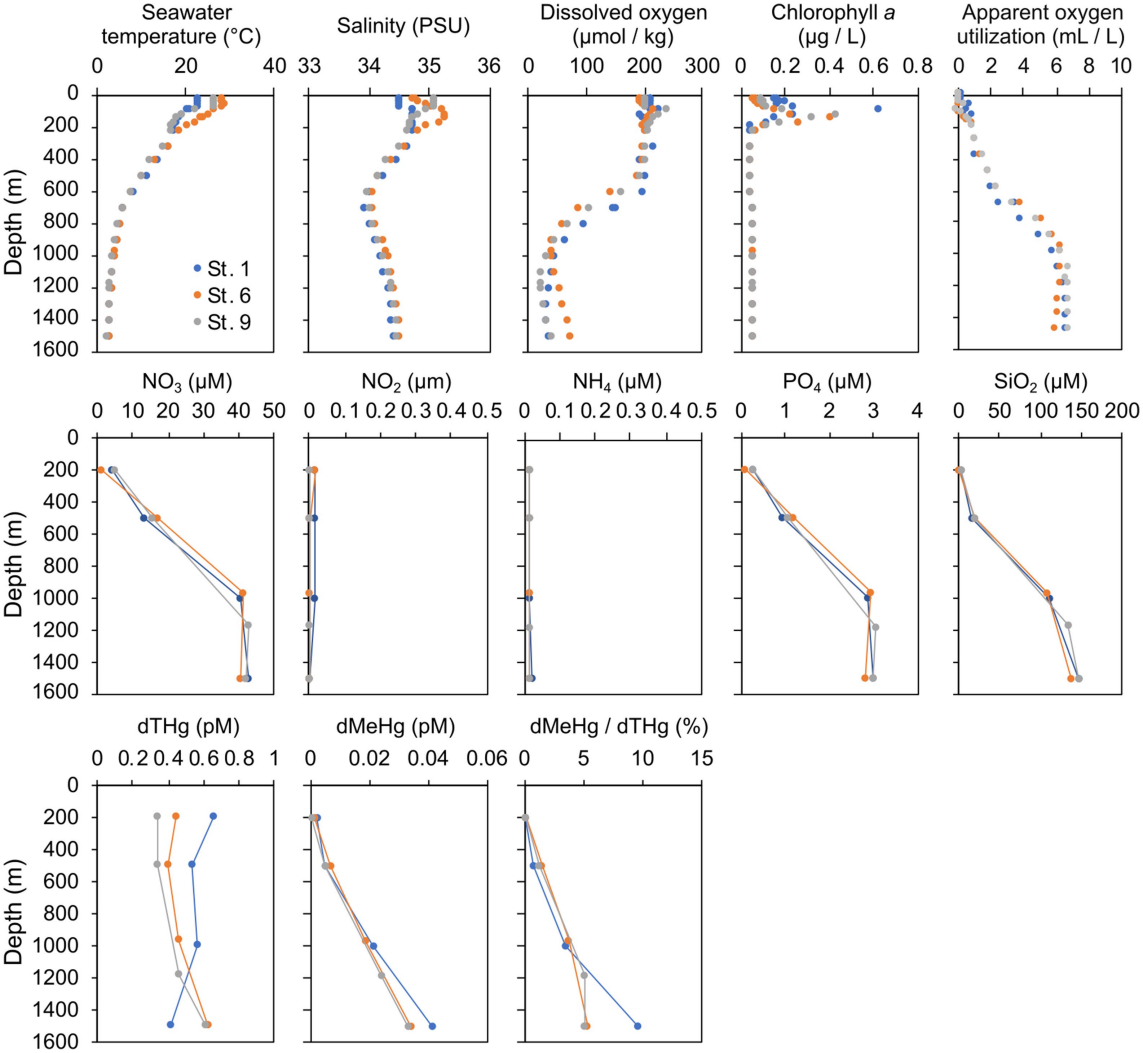
Depth profiles of environmental factors, including macronutrients and dissolved mercury (Hg) and methylated mercury (MeHg) concentrations in the western North Pacific Subtropical Gyre.

The seawater temperature and dissolved oxygen concentration decreased from the surface to the mesopelagic layer (Figure 2). The depth of the oxygen minimum zone at each station was 1,000 m at St. 1, 967 m at St. 6, and 1,175 m at St. 9, respectively. In contrast, the nitrate, phosphate, and silicate concentrations increased with increasing depth. dTHg concentrations ranged from 0.42 to 0.66 at St. 1, from 0.46 to 0.63 at St. 6, and from 0.40 to 0.62 pM. The dMeHg concentrations and their proportion of dTHg increased with depth at all stations. The dMeHg concentration at 1,500 m deep was 0.033–0.041 pM, representing 5.2–9.8% of the dTHg. dMeHg concentration was significantly positively correlated with NO_3_, PO_4_, SiO_2_ concentrations, and AOU (*p* < 0.01, *n* = 12). In contrast, no correlation was observed between dTHg concentrations and environmental parameters.

### 3.2 Abundance and distribution of prokaryotes, *rpoB*, and Hg-related genes

**Figure 3:**
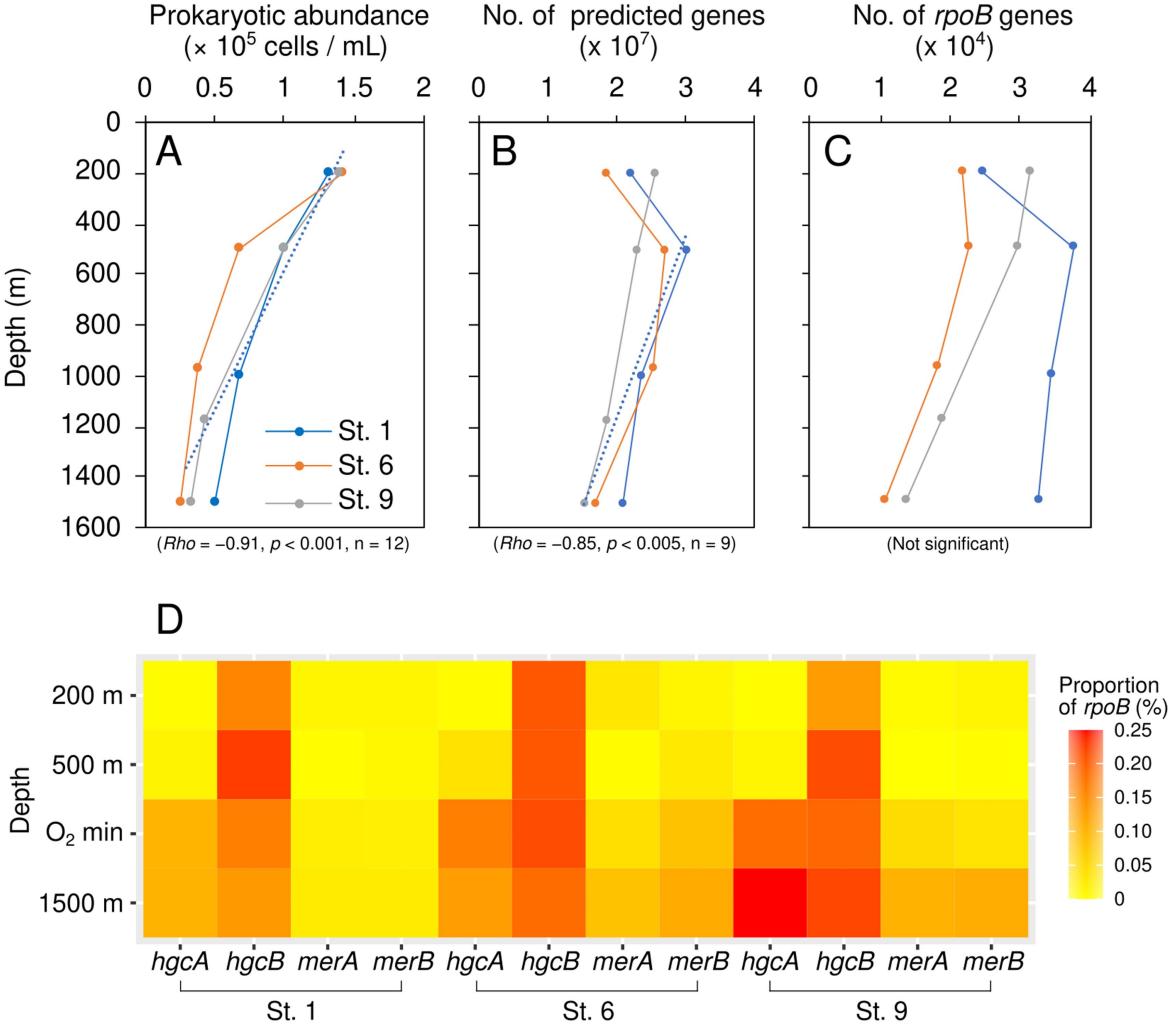
Depth profiles of prokaryotic abundance **(A)**, total predicted genes **(B)**, and *rpoB* genes **(C)**, and the depth distribution of Hg-related genes shown as a heatmap **(D)**.

The prokaryotic abundance decreased with increasing depths and ranged from 0.52 × 10^5^ to 3.56 × 10^5^ cells mL^−1^ at St. 1, 0.29 × 10^5^ to 3.95 × 10^5^ cells mL^−1^at St. 6, and from 0.35 × 10^5^ to 4.19 × 10^5^ cells mL^−1^ at St. 9 (*Rho* = −0.91, *p* < 0.001, *n* = 12) (Figure 3A). The number of predicted genes decreased below 500 m depth (*Rho* = −0.85, *p* < 0.005, *n* = 9) (Figure 3B). The abundance of the *rpoB* gene also tended to decline below 500 m, but this trend was not statistically significant (Figure 3C). The number of *hgcA*, *merB*, and *merA* genes tended to increase in the mesopelagic layers (from oxygen minimum zone to 1,500 m depths), whereas this trend was not observed for *hgcB* (Figure 3D). The relative abundance of *hgcA*, *merB*, and *merA* in the mesopelagic layers ranged from 0.10 to 0.25%, from 0.02 to 0.11, and from 0.02 to 0.10% for *rpoB*. Detailed information regarding metagenome sequences, contigs, predicted genes, and Hg-related genes is shown in Supplementary Table S1.

**Figure 4:**
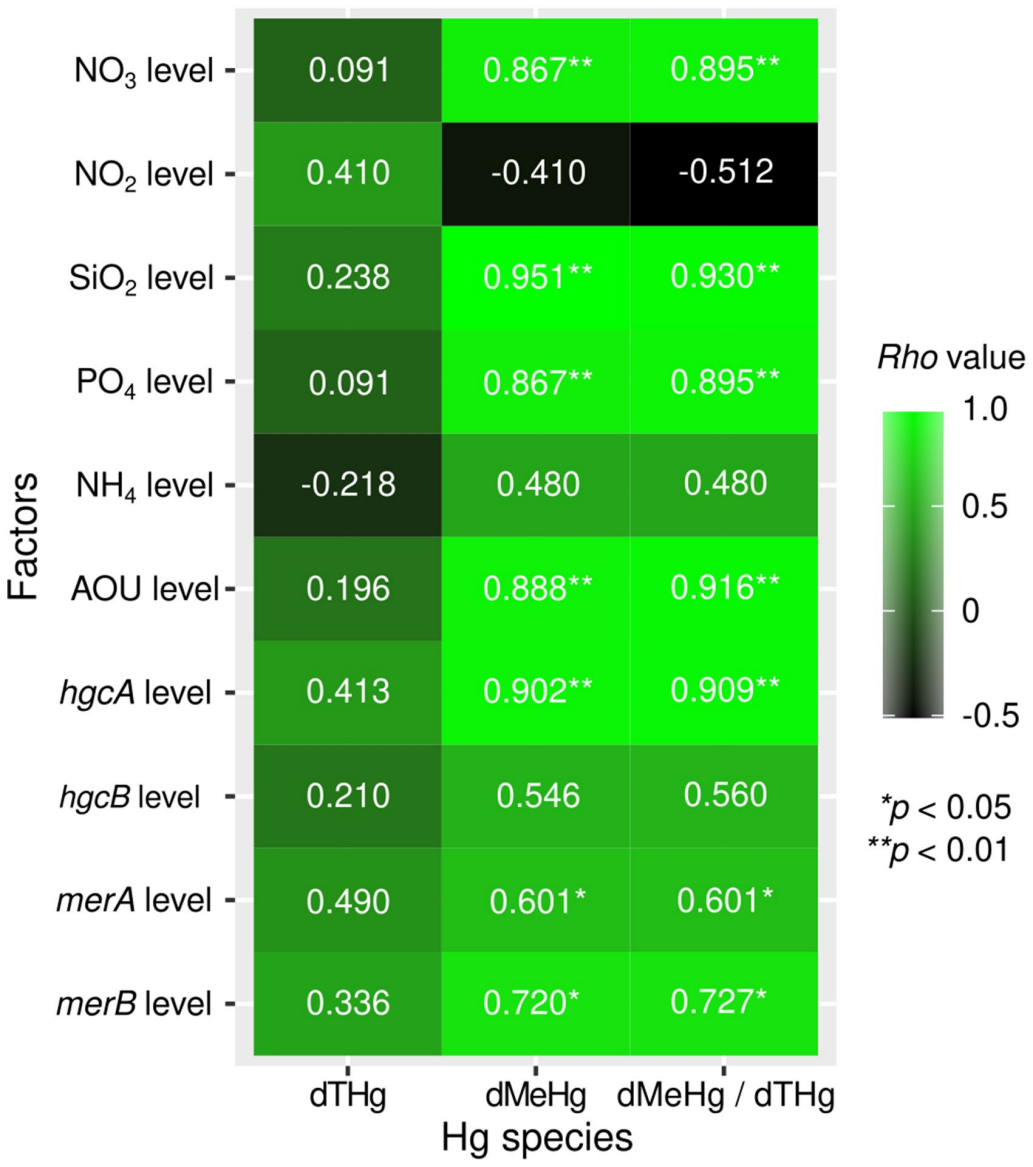
Heatmap showing Spearman’s rank correlation coefficients between total mercury (THg) or methylmercury (MeHg) concentrations and environmental factors.

A significantly positive correlation was observed between *hgcA* relative abundance and dMeHg concentration, as well as the dMeHg / dTHg ratio (*p* < 0.01, *n* = 12) (Figure 4). In addition, a significantly positive correlation was noted between dMeHg and *merB* (*p* < 0.05, *n* = 12). A significant correlation was also observed between *hgcA* and *merB* abundance and NO_3_, PO_4_, and SiO_2_, and AOU (*p* < 0.01, *n* = 12). These results suggest that the presence of Hg-related genes in prokaryotes influences the distribution of MeHg in seawater, which is in turn affected by various environmental factors. The *hgcA* abundance was higher than that of *merB* and *merA*, with *merA* being slightly more abundant than *merB* (Supplementary Figure S3).

### 3.3 Phylogenies and depth distribution of the Hg-related genes

**Figure 5:**
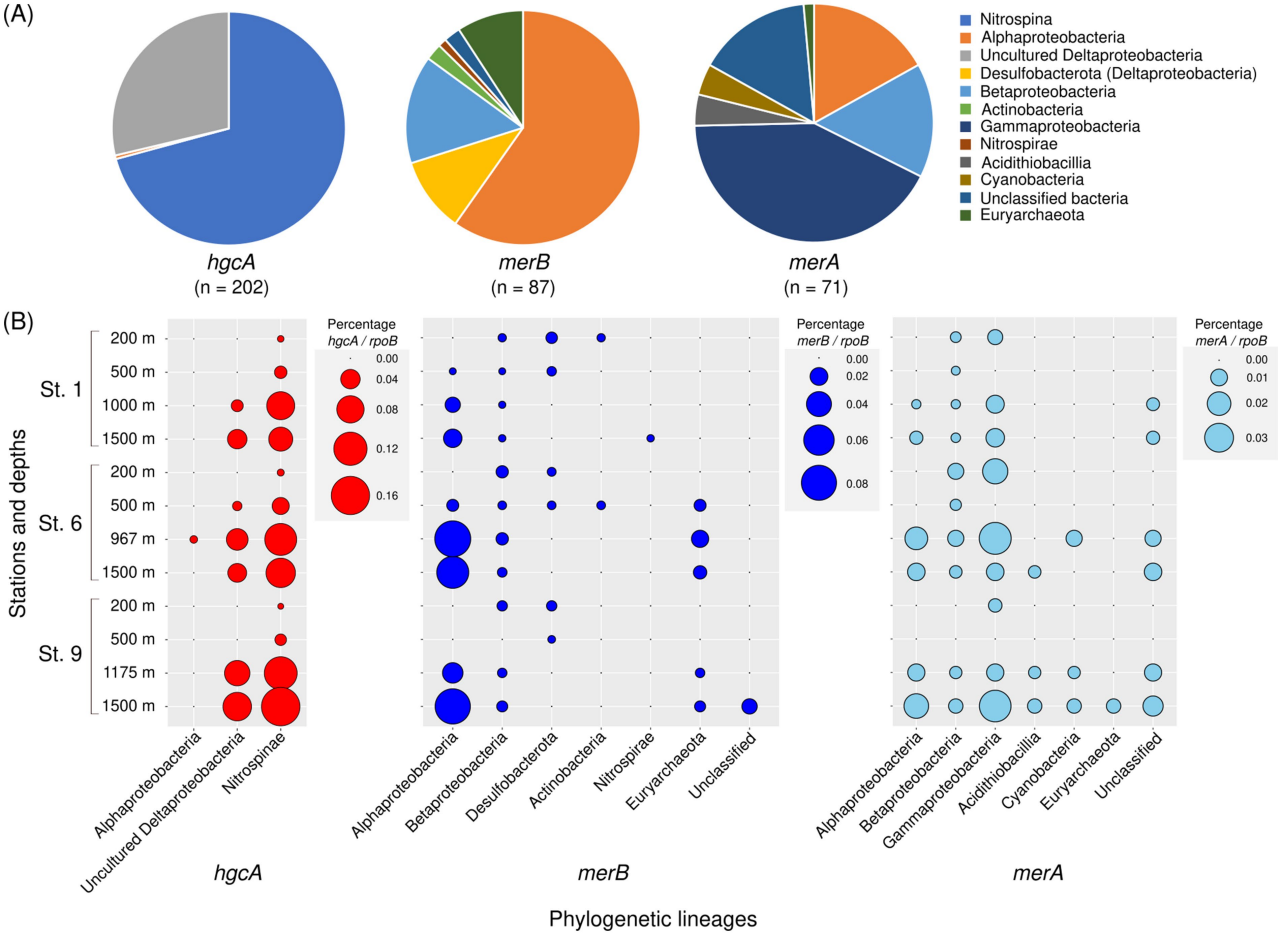
Proportion of phylogenetic lineages in *hgcA*, *merB*, and *merA* sequences detected in the survey area **(A)** and depth distribution of each phylogenetic lineage at each sampling station **(B)**.

Phylogenetic analysis of *hgcA* (methyl carrier), *merB*, and *merA* indicated that a distinct phylogenetic lineage was dominant for each Hg-related gene (Figure 5A and Supplementary Figures S4–S7). *Nitrospina* dominated the total *hgcA*-like sequences and accounted for 70.8% of *hgcA* genes. This lineage was predominant among the *hgcB* genes (546 out of 550 sequences) (Supplementary Figure S5 and Supplementary Table S2). Additional *hgcA* genes were affiliated with Uncultured Deltaproteobacteria and Alphaproteobacteria, representing 28.7 and 0.5% of *rpoB* genes, respectively. In *merB*, various lineages were identified, including Alphaproteobacteria (59.8%), Betaproteobacteria (14.9%), Deltaproteobacteria (10.3%), Euryarchaeota (9.2%), Actinobacteria (2.3%), Nitrospirae (1.1%), and unclassified bacteria (2.3%). Alphaproteobacteria and Betaproteobacteria were also detected in *merA* and accounted for 16.9 and 15.5% of *rpoB* genes, respectively. However, Gammaproteobacteria (42.3%) was the dominant lineage in total *merA* sequences. Additionally, Acidithiobacillia, Cyanobacteria, and unclassified bacteria *merA* were also observed.

The vertical distribution of each phylogenetic lineage associated with *hgcA*, *merB*, and *merA* is shown in Figure 5B and Supplementary Figures S3–S7. *Nitrospina* and uncultured Deltaproteobacteria *hgcA* sequences were abundant in the mesopelagic layers at all stations. Alphaproteobacteria *merB* were observed below 500 m and were abundant at a depth of 1,500 m at all stations. Additionally, Euryarchaeota *merB* sequences were detected between 500 m and 1,500 m. In contrast, *merB* sequences affiliated with Desulfobacterota and Actinobacteria were detected in the upper 500 m. Notably, Betaproteobacteria *merB* was widely distributed from the surface to mesopelagic layers. For the *merA* genes, Betaproteobacteria and Gammaproteobacteria sequences were distributed from the surface to mesopelagic layers. The abundance of Gammaproteobacteria *merB* increased at 1,500 m deep at Sts. 6 and 9. *MerA* sequences affiliated with Actinobacteria and Cyanobacteria were detected below 500 m. Similar to the distribution of *merB*, Alphaproteobacteria *merA* were abundant in the mesopelagic layers across all stations.

### 3.4 Metabolic functions of MAGs with Hg-related genes

**Figure 6:**
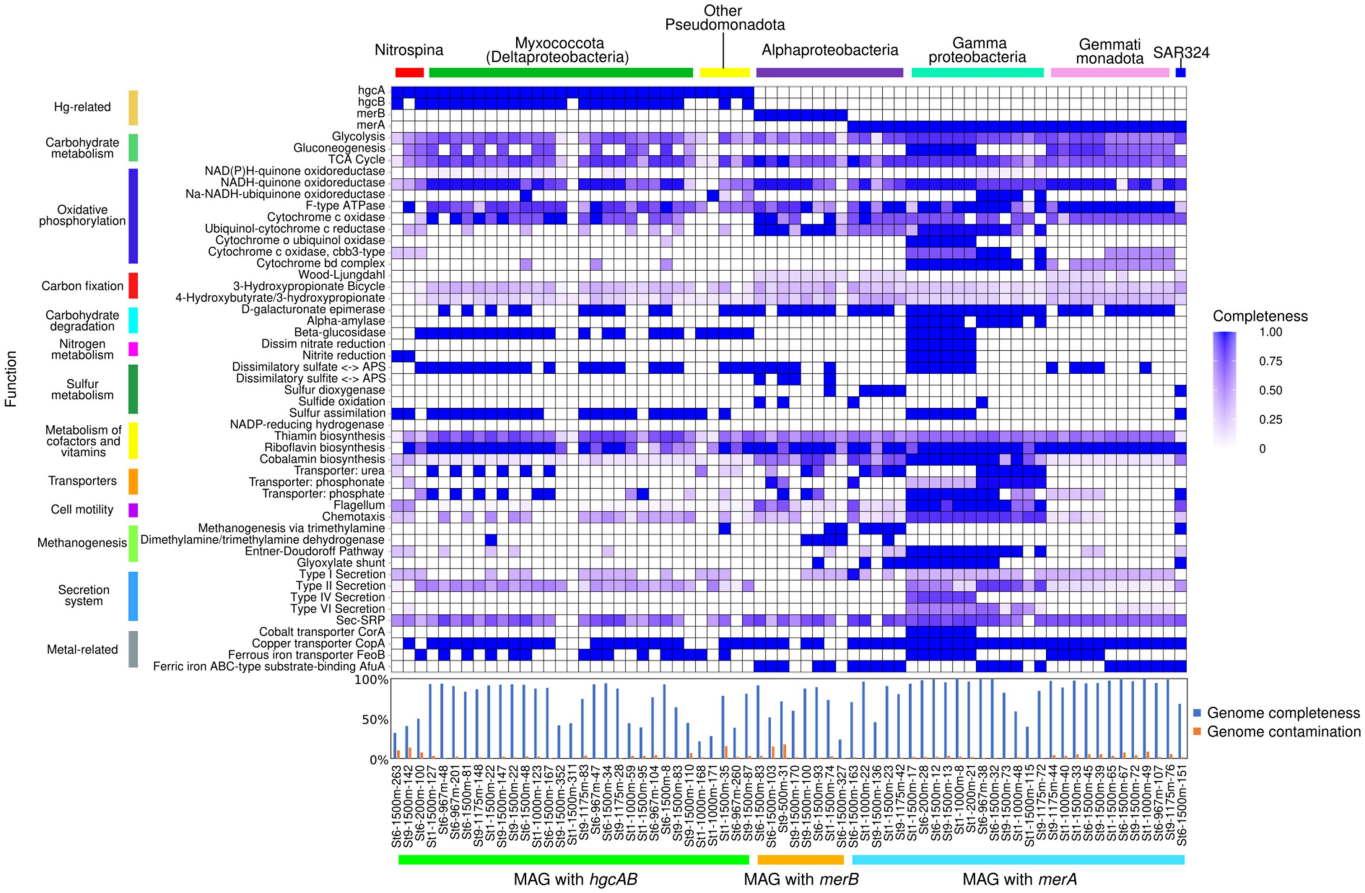
Metabolic pathways of metagenome-assembled genomes (MAGs) with Hg-related genes. Names, genome completeness, and contamination of MAGs with *hgcAB*, *merB*, and *merA* are represented at the bottom of the heatmap. Their phylogenetic lineages are shown at the top of the heatmap. Metabolic pathways are represented on the left side of the heatmap. Color gradations of each cell refer to the completeness of pathway modules. The completeness and contamination rate of MAG are shown in the bar graph.

A total of 1,308 MAGs was constructed from metagenome sequences (Table 2). Pathway analysis revealed that MAGs carrying *hgcAB*, *merB*, and *merA* contained several distinctive metabolic functions (Figure 6). *hgcAB*-carrying *Nitrospina* possessed the cytochrome c oxidase and nitrite reduction pathways; however, their genome completeness values were relatively low (30–50%). MAGs affiliated with *Myxococcota* (Deltaproteobacteria) harboring *hgcAB* included genes for dissimilatory sulfate in the adenosine phosphosulfate pathway. *Myxococcota* and other *Pseudomonadota* MAGs with *hgcAB* also exhibited genes involved in beta-glucosidase, dissimilatory sulfate, and sulfur assimilation pathways. *merB*-carrying MAGs affiliated with Alphaproteobacteria contained genes involved in methanogenesis via trimethylamine. Additionally, genes associated with dissimilatory sulfite to adenosine phosphosulfate and sulfide oxidation pathways were observed in an alphaproteobacterial MAG with *merB*. Gammaproteobacteria MAGs with *merA* exhibited versatile metabolic pathways such as oxidative phosphorylation, carbohydrate metabolism, nitrogen metabolism, sulfur metabolism, methanogenesis, and secretion systems. In contrast, Gemmatimonadota MAGs showed comparatively simple metabolic profiles, comprising carbohydrate metabolism, oxidative phosphorylation, sulfur metabolism, cofactor and vitamin metabolism, and metal-related transporters. Metabolic pathways related to methane and sulfur cycles were observed in a SAR324 MAG.

**Table 2:** Number of metagenome-assembled genomes (MAGs) constructed from metagenome sequences.

No. of MAGs	St. 1				St. 6				St. 9			
	200 m	500 m	1,000 m	1,500 m	200 m	500 m	967 m	1,500 m	200 m	500 m	1,175 m	1,500 m
Total	115	103	61	83	136	157	114	114	136	96	99	94
*hgcAB*+	0	0	4	4	1	0	5	7	0	0	3	7
*merB+*	0	0	0	1	0	0	0	4	0	1	0	2
*merA+*	1	0	5	5	1	0	2	6	0	0	4	5

## 4 Discussion

### 4.1 Potential of microbial Hg methylation and demethylation in the seawater column of the WNPSG

In the upper mesopelagic layers in the WNPSG, a significant positive relationship was observed between dMeHg (dMeHg/dTHg ratio) and AOU. Generally, the AOU serves as a proxy for microbial activity, suggesting that prokaryotes within the seawater column possibly contribute to MeHg production in the WNPSG. Similar positive correlations between MeHg concentration and AOU in the seawater column were observed in the upper mesopelagic layers (<1,500 m depth) in the Atlantic Ocean (Mason and Sullivan, 1999), the Mediterranean Sea (Cossa et al., 2009; Cossa et al., 2011), and the Oyashio and Kuroshio regions of the western North Pacific (Tada et al., 2021). Notably, the WNPSG is a subtropical gyre characterized by relatively low nutrient supply and reduced vertical organic matter flux (Karl and Church, 2017). The persistence of a clear correlation between dMeHg (or dMeHg/dTHg) and AOU even under these low-productivity conditions suggests that MeHg production driven by local remineralization can be significant even in areas with diffident organic matter input. Furthermore, the linkage between oxygen consumption and Hg methylation is a robust feature across contrasting oceanic environmental conditions. Additionally, the relative abundance of *hgcA* increased in the oxygen-depleted mesopelagic layers with high dMeHg concentrations (*hgcB* was also detected). Taken together, WNPSG data and observations from the Atlantic, Mediterranean, North Pacific marginal seas, and Black Sea (Villar et al., 2020; Tada et al., 2021; Cabrol et al., 2023) suggest that *hgcA*-possessing prokaryotes (strictly speaking, both *hgcAB*-possessing) contribute to the formation of relatively high MeHg-containing water masses in oxygen-depleted mid-water layers across a range of environments, from highly productive to oligotrophic.

The depth profile of *hgcB* relative abundance differed from that of *hgcA*, and no positive correlation was observed between *hgcB* and MeHg concentration. These discrepancies in the distribution of *hgcAB* have been observed in the Arctic Ocean metagenomes (Bowman et al., 2020) and in previous studies conducted in the East China Sea and the Oyashio region (Tada et al., 2021). Detection efficiencies for *hgcB* may be limited by protocol and assembly methods optimized primarily for *hgcA* detection. Similarly, differences in the evolutionary background and conservation between *hgcA* and *hgcB* could lead to discrepancies in their detection rates in metagenomic analyses. These findings highlight the need to consider both methodological and evolutionary factors when interpreting the prevalence of Hg-methylating genes in environmental samples.

In a metagenomic survey of Hg-related genes, *merB* was detected in the mesopelagic layers of both the Arctic Ocean and the equatorial Pacific (Bowman et al., 2020). Furthermore, incubation experiments using enriched Hg isotopes demonstrated that MeHg demethylation can occur within the central Pacific water column (Munson et al., 2018), indicating that microbial MeHg demethylation may co-occur with Hg methylation in mesopelagic zones. In the WNPSG, an increased relative abundance of *merB* in high-dMeHg mesopelagic layers further supports the widespread distribution of *merB*-carrying prokaryotes and their potential involvement in MeHg degradation. A positive correlation between *merB* abundance and dMeHg concentration suggests that in environments rich in methylmercury, microorganisms possessing MeHg degradation capabilities may exist as a defense mechanism against it. Notably, the higher relative abundance of *hgcA* than that of *merB* in the WNPSG implies that Hg methylation is more prominent than MeHg demethylation in these layers. Although Bowman et al. (2020) reported that *merB* abundance exceeded that of *hgcA* in the equatorial North Pacific (8°N, 156°W), these samples were collected from depths of 800 m or less, complicating direct comparisons with deeper profiles.

### 4.2 Phylogeny and function of Hg methylators in the WNPSG

Phylogenetic analysis of Hg-related genes supports previous findings that *Nitrospina* bacteria may be the dominant Hg methylators in the mesopelagic layers of the open ocean (Bowman et al., 2020; Villar et al., 2020; Tada et al., 2020, 2021). Metatranscriptomic data from the Tara Oceans project confirmed *hgcA* expression in this lineage (Villar et al., 2020), indicating a widespread distribution and potential contribution of *Nitrospina* bacteria to Hg methylation on a basin scale. However, the actual Hg methylation capacity of the *Nitrospina* lineage remains unconfirmed, as no isolates harboring *hgcA* have been obtained. For example, the genome of *Nitrospina gracilis* is associated with nitrite oxidation through the presence of genes encoding ferredoxin-nitrite reductase, copper-containing nitrite reductase, and nitrite oxidoreductase (Lücker et al., 2013). Additionally, *Nitrospina* lineages have been detected in oxygen minimum zones (Spieck et al., 2014; Pachiadaki et al., 2017; Sun et al., 2019), and their MAGs include cytochrome c oxidase, suggesting adaptation to low-oxygen environments (Sun et al., 2019). Functional pathway analysis further revealed that *Nitrospina* MAGs harboring *hgcAB* also possess nitrite-reducing pathways and cytochrome c oxidase, supporting their potential role in Hg methylation under low-oxygen conditions. Additionally, studies employing enriched Hg isotopes have identified a positive correlation between Hg methylation and nitrification rates (Starr et al., 2022; Despins et al., 2023), suggesting a close relationship between microbial Hg methylation by *Nitrospina* bacteria and the oceanic nitrogen cycle.

Members of Deltaproteobacteria, the second dominant lineage among *hgcA*-carrying microbes in the metagenome, have been identified in oxygen-deficient seawater in coastal seawater (Capo et al., 2020; Lin et al., 2021; Tada et al., 2023) as well as in the open ocean (Villar et al., 2020). In the WNPSG, *hgcA* affiliated with uncultured Deltaproteobacteria were particularly abundant. Distinct deltaproteobacterial *hgcA* sequences associated with *Desulfovibrionales*, *Desulfobacterales*, and *Syntrophobacterales* were observed in the Tara Oceans metagenomes (Villar et al., 2020), suggesting that diverse deltaproteobacterial lineages are involved in Hg methylation in the open ocean environments. Moreover, Deltaproteobacteria-related MAGs closely related to Myxococcota contained genes for dissimilatory sulfate reduction, indicating a potential link between Hg methylation and the sulfur cycle in the mesopelagic layers of the WNPSG.

*Nitrospina hgcA* and Deltaproteobacteria *hgcA* were frequently detected in the mesopelagic layer, but 16S rRNA gene analysis showed no significant increase in the relative abundance of *Nitrospina* or Deltaproteobacteria in these layers (Supplementary Figures S8, S9). Notably, the relative abundance of *Nitrospina* tended to be higher in the upper mesopelagic layer (200–500 m). These findings suggest that specific *Nitrospina* and Deltaproteobacteria, which cannot be identified solely from 16S rRNA gene analysis, contribute to Hg methylation within the WNPSG.

To date, Hg methylation by aerobic microorganisms under aerobic conditions also remains unconfirmed. However, one *hgcA* gene affiliated with Alphaproteobacteria and closely related to *Defluviimonas indica—*a strictly aerobic and chemoheterotrophic marine bacterium (Jiang et al., 2014)—was reported in the mesopelagic layer, suggesting that aerobic microbes may be involved in Hg methylation in the open ocean. Although metagenomic approaches can identify the potential for Hg methylation, culture-based research is necessary to confirm methylation activity. Therefore, combining culture-dependent and independent methods is essential to fully elucidate the processes underlying microbial MeHg production in marine environments.

### 4.3 Phylogeny and function of MeHg demethylators and Hg reducers in the WNPSG

The phylogenetic distribution of *merB* is more limited than that of *merA* (Christakis et al., 2021). However, diverse lineages of *merB* were detected in the mesopelagic layers of the WNPSG, similar to *merA*. Notably, phylogenetic lineages with both *merB* and *merA* are likely crucial in removing MeHg from the mesopelagic zone. In the present study, members of *Novosphingobium* (Alphaproteobacteria) were detected in both *merB* and *merA* gene datasets, suggesting their potential to transform MeHg to Hg(0). Unfortunately, no MAGs containing both *merB* and *merA* genes were detected, likely because of limited sequence depth. Thus, additional metagenomic reads are necessary to clarify the metabolic function of these lineages.

Notably, some alphaproteobacterial MAGs harboring either *merB* or *merA* also possessed pathways for methanogenesis via trimethylamine (TMA), aligning with previous observations of elevated methane concentrations in the anoxic layers in the eastern North Pacific (Thamdrup et al., 2019). This finding suggests that microbial processes involved in both MeHg degradation and Hg(II) reduction may be coupled with methane production in the mesopelagic zone.

*Burkholderia* (Betaproteobacteria), a lineage carrying both *merB* and *merA* sequences, was detected throughout the water column, from the surface to mesopelagic layers. Although typically rare in marine environments (Morris et al., 2006), *Burkholderia* accounted for 0.02–2.1% (average 0.39%) of total sequences (Supplementary Figure S9), suggesting that this low-abundance lineage may nonetheless play a crucial role in MeHg degradation and Hg reduction in the mesopelagic layers of the WNPSG.

Contrary to earlier culture-dependent studies, which failed to identify bacteria possessing *merB* without *merA* (Boyd and Barkay, 2012), the findings of this study show that certain lineages, including Desulfobacterota (Deltaproteobacteria) and Nitrospirae possess only *merB*. These organisms may use alternative pathways for Hg(II) reduction, as some Alphaproteobacteria (e.g., *Magnetospirillum gryphiswaldense* MSR-1 and *M. magnetotacticum* MS-1) lack *merA* reduce Hg(II) to Hg(0) (Liu and Wiatrowski, 2018). A genomic survey further revealed that nearly half of the prokaryotic genomes with *merB* lack *merA* (Christakis et al., 2021). Although the Hg(II) reduction capacity of the *merB*-only lineages detected in this study remains unconfirmed, they may contribute to MeHg degradation in the WNPSG.

A survey of Hg-related genes in ocean genome databases suggests the widespread marine distribution of *merA*-carrying microorganisms (Sanz-Sáez et al., 2022). In this study, Gammaproteobacteria dominated *merA*-containing lineages from the surface to mesopelagic layers, with *Marinobacter* (Alteromonadales) representing the most abundant group (14 out of 38 sequences). *Marinobacter merA* was consistently detected across depths, and 16S rRNA gene sequencing revealed increased relative *Marinobacteraceae* abundance in mesopelagic layers (Supplementary Figure S9). Given that 89.1% of tested Marinobacter isolates from surface to deep-sea environments harbor *merA* genes (Sanz-Sáez et al., 2022), these findings suggest that *Marinobacter* plays a key role in Hg(II) reduction in mesopelagic waters globally.

Metagenomic and metatranscriptomic surveys across the Atlantic, Pacific, and Indian Oceans have identified diverse *merA*-harboring lineages, including *Corynebacteriales* (Actinomycetota), *Rhodobacterales* (Alphaproteobacteria), *Alteromonadales*, *Oceanospirillales*, *Moraxellales* (Gammaproteobacteria), and *Flavobacteriales* (Bacteroidota) (Sanz-Sáez et al., 2024). This study additionally detected *Burkholderia* (Betaproteobacteria), *Sphingobium* (Alphaproteobacteria), *Halothece* (Cyanobacteria), and *Methanosarcinales* (Euryarchaeota), highlighting the broad taxonomic diversity of *merA*-containing microorganisms in marine environments. Furthermore, *merA* sequences related to Betaproteobacteria have also been reported in the coastal waters of Japan (Tada et al., 2023), suggesting their potential role in Hg(II) reduction across both coastal and open ocean ecosystems.

Metabolic pathway analysis revealed that Alphaproteobacterial MAGs containing either *merB* or *merA* possess a TMA-linked methane production pathway. Although TMA concentrations in seawater are typically low (nanomolar levels), elevated levels near the thermocline and oxycline have been reported (Gibb et al., 1999; Cree et al., 2018). In coastal sediments, benthic animals and phytoplankton serve as major TMA sources through direct release or decomposition (Wang and Lee, 1994). These findings suggest that TMA-driven methane production may support concurrent MeHg demethylation and Hg(II) reduction in the mesopelagic layers of the WNPSG.

Gemmatimonadota, a rare lineage in marine environments (<1% of 16S rRNA sequences) (Aldeguer-Riquelme et al., 2023), demonstrates metabolic versatility, including organic carbon degradation, denitrification, sulfate reduction, and sulfide oxidation (Gong et al., 2024). In this study, 11 out of 36 *merA*-carrying MAGs were affiliated with Gemmatimonadota. Additionally, the SAR324 lineage, which has diverse metabolic potential spanning heterotrophic and autotrophic pathways (Malfertheiner et al., 2022), also contained *merA*, with pathways for carbohydrate, sulfur, and metal metabolism. Although Hg(II) resistance in these groups remains unconfirmed, their metabolic traits suggest a role in Hg speciation associated with carbon and sulfur cycling in marine ecosystems.

In conclusion, the study investigated Hg methylation and MeHg demethylation processes mediated by microorganisms in the WNPSG water column. A significant positive correlation between MeHg concentration and AOU suggests that microbial activity (presumably microbial remineralization of organic matter) plays a crucial role in Hg methylation. The abundance of *hgcA*, a key gene for Hg methylation, increased in oxygen-depleted mesopelagic layers with high MeHg concentrations. *merB*, involved in MeHg demethylation, was also prevalent in the WNPSG, indicating simultaneous MeHg degradation. However, the relatively higher abundance of *hgcA* compared to *merB* suggests that Hg methylation processes may dominate over demethylation. Phylogenetic analysis of *hgcA* sequences identified *Nitrospina* bacteria with nitrite reductase pathway as dominant Hg methylators, linking Hg methylation to the nitrogen cycle. Additionally, *Myxococcota* (Deltaproteobacteria) was associated with sulfur cycling. Diverse microbial lineages carried *merB* and *merA*, suggesting co-occurring MeHg demethylation and Hg(II) reduction in the mesopelagic zone. Gammaproteobacteria, particularly *Marinobacter*, emerged as key contributors to Hg reduction. These results highlight the complex microbial interactions driving Hg transformations in the mesopelagic layers in the WNPSG. Functional pathway analysis of MAGs with Hg-related genes provides insight into microbial Hg transformations in the ocean. However, future studies integrating comprehensive datasets of *hgcA*, *merB*, and MeHg concentrations are essential to clarify the depth-related distribution of MeHg and associated microbial processes.

### 4.4 High-confidence screening of *merA* and *merB* genes by sequence signature

In this study, we adopted a conservative residue-based curation strategy to identify *merA* and *merB* homologs in our metagenomes. As detailed in the ‘Materials and Methods’ section, we excluded *merA* sequences lacking any of the following conserved residues present in the *Bacillus* sp. RC607 MerA reference: the catalytic cysteine pair Cys-207 and 212; Tyr-or Phe-605; and the C-terminal vicinal Cys-628 and 629. Similarly, merB sequences were discarded if they lacked Cys-96, Asp-99, Cys-117 and Cys-159, which together form a catalytic and structurally important cysteine cluster in the R831b MerB reference. By restricting our analyses to sequences that retain the full set of experimentally validated catalytic and metal-binding residues, we focused on gene variants most likely to encode functional MerA and MerB enzymes, reducing the impact of spurious annotations, partial open reading frames and distant paralogues. This stringent curation unavoidably leads to conservative estimates of *merA* and *merB* prevalence because atypical variants lacking one or more of these residues were not considered. While some of these variants may represent nonfunctional remnants, others could encode enzymes with altered catalytic properties or substrate spectra. Therefore, our counts should be regarded as minimum estimates of canonical, biochemically supported *merA* and *merB* genes. Future work combining targeted biochemical characterization with metagenomic surveys will be required to clarify the ecological roles of these atypical variants.

## Data Availability

The datasets presented in this study can be found in online repositories. The names of the repository/repositories and accession number(s) can be found in the article/Supplementary material.

## Footnotes

1
https://qiime.org/

2
https://www.kegg.jp/ghostkoala/

3
https://github.com/bjtully/BioData/blob/master/KEGGDecoder/README.md
