# Supplementary material for: Distribution and function of prokaryotes involved in mercury methylation, demethylation, and reduction in the western North Pacific Subtropical Gyre
Source: Front Microbiol. 2026 Jan 22;16:1642479. doi: 10.3389/fmicb.2025.1642479 (PMC12874090; doi:10.3389/fmicb.2025.1642479)
Supplement: Supplementary file 10 [file Presentation_1.PPTX]

## Slide 1
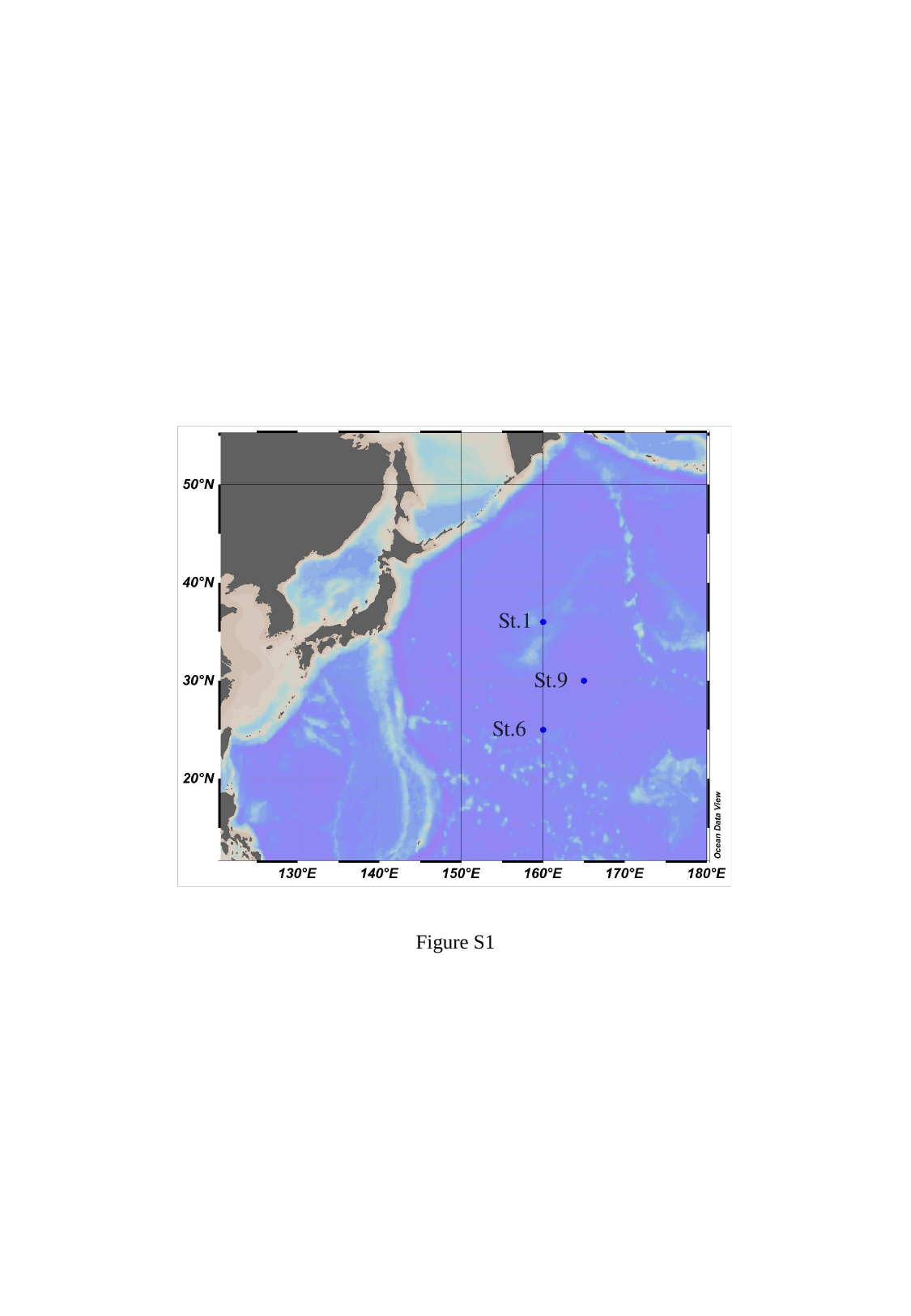

Figure S1
Figure S1

## Slide 2
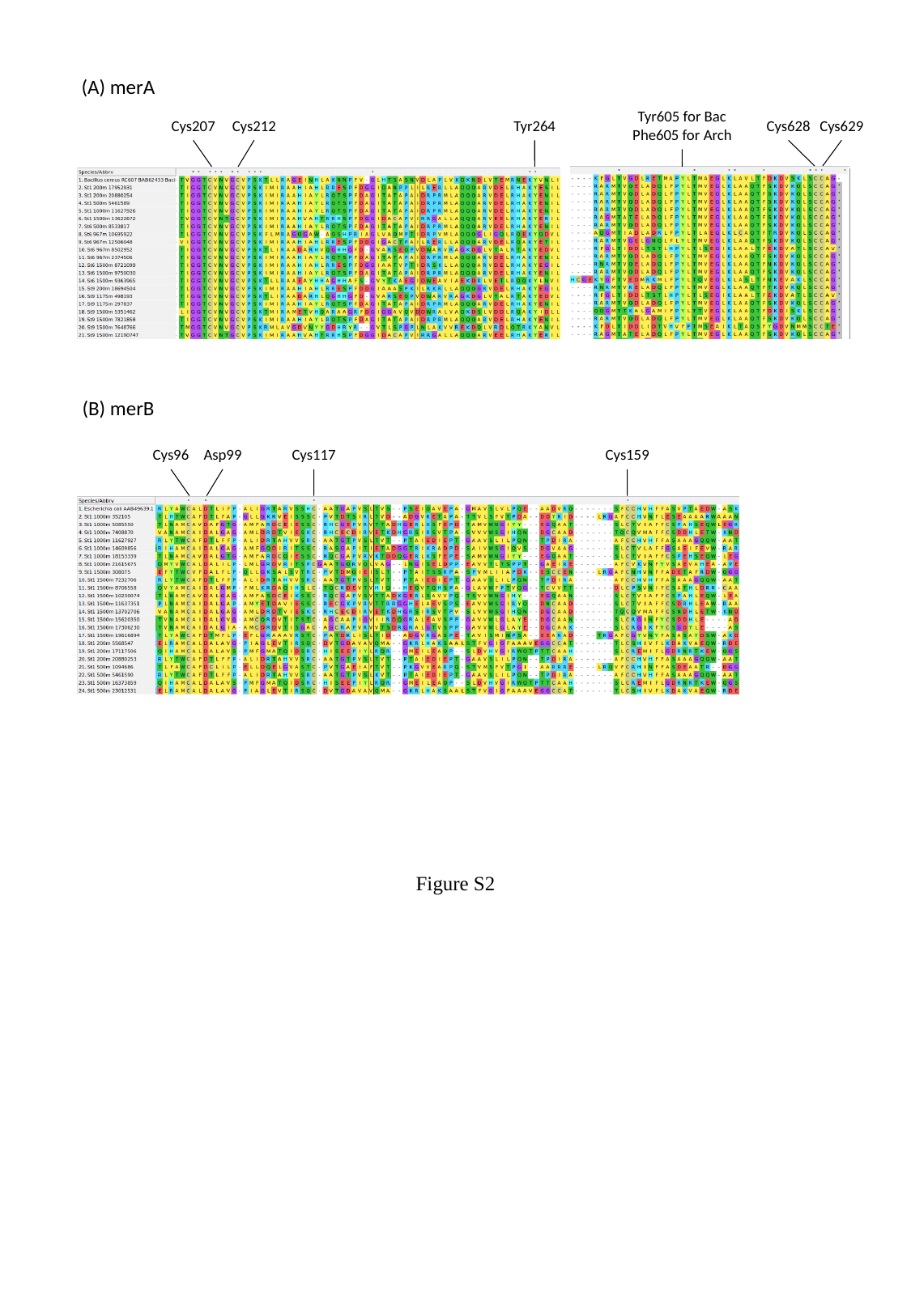

(A) merA
Tyr605 for Bac
Phe605 for Arch
Cys207
Cys212
Tyr264
Cys628
Cys629
(B) merB
Cys96
Asp99
Cys117
Cys159
Figure S2

## Slide 3
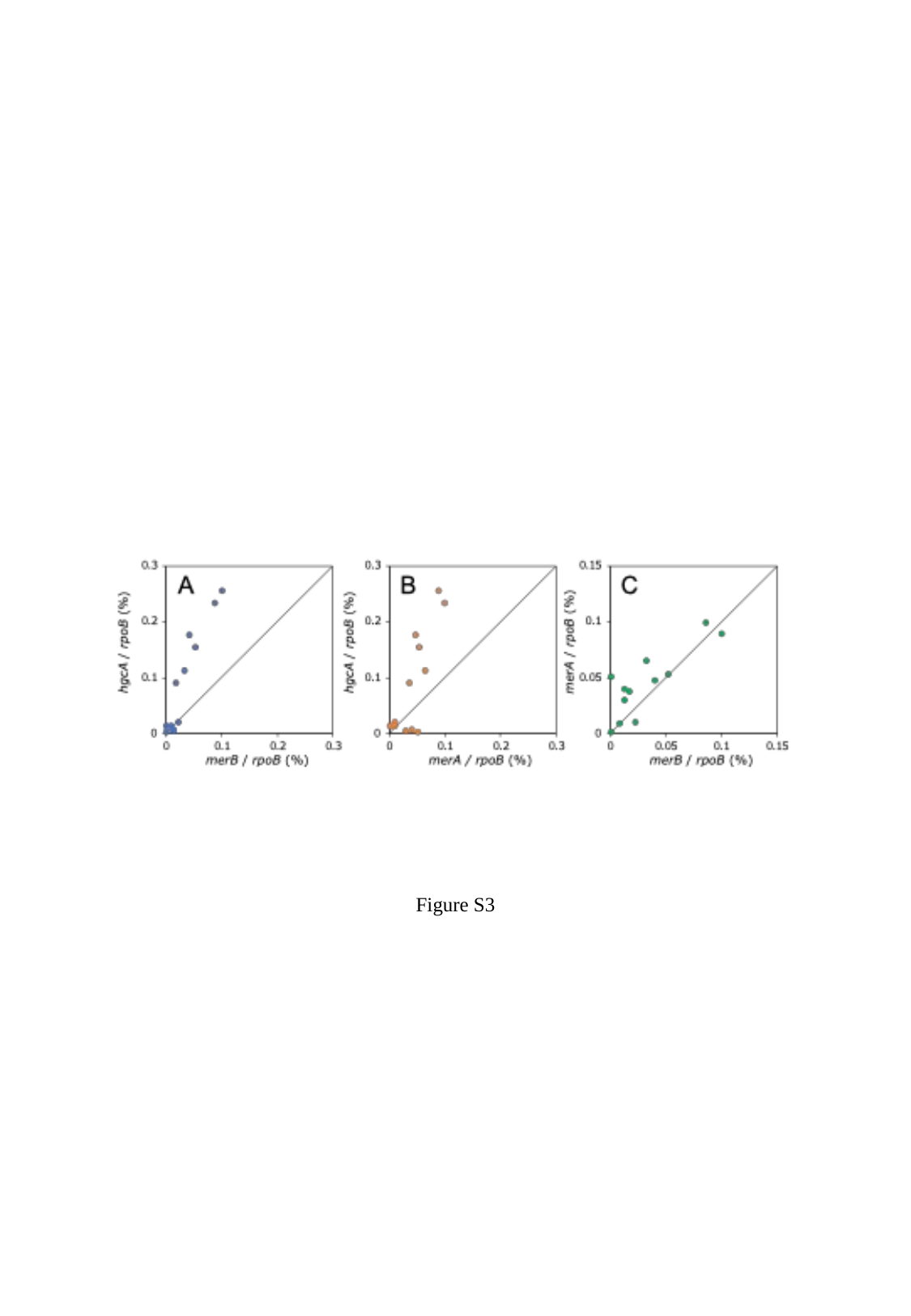

Figure S3

## Slide 4
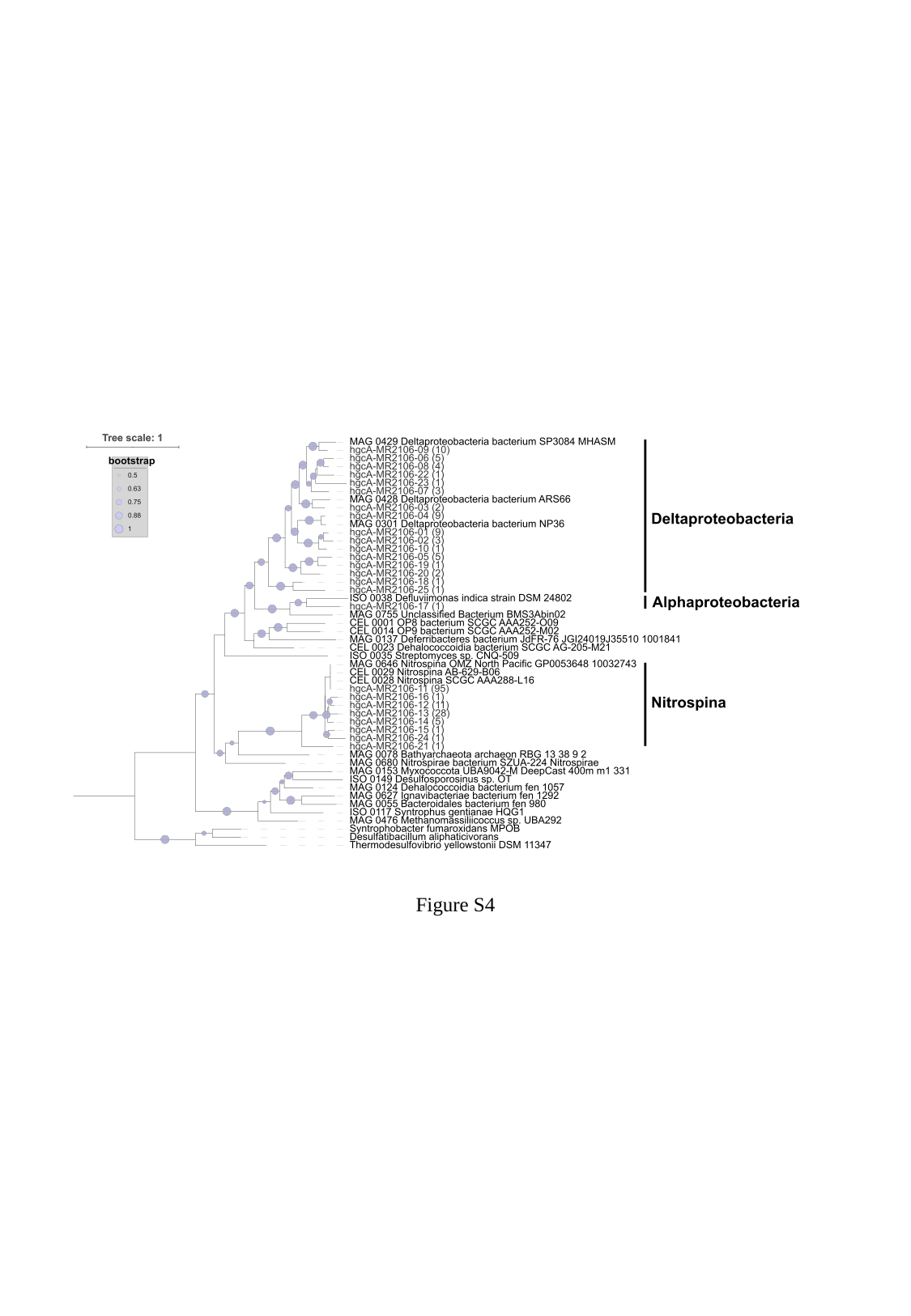

Figure S4

## Slide 5
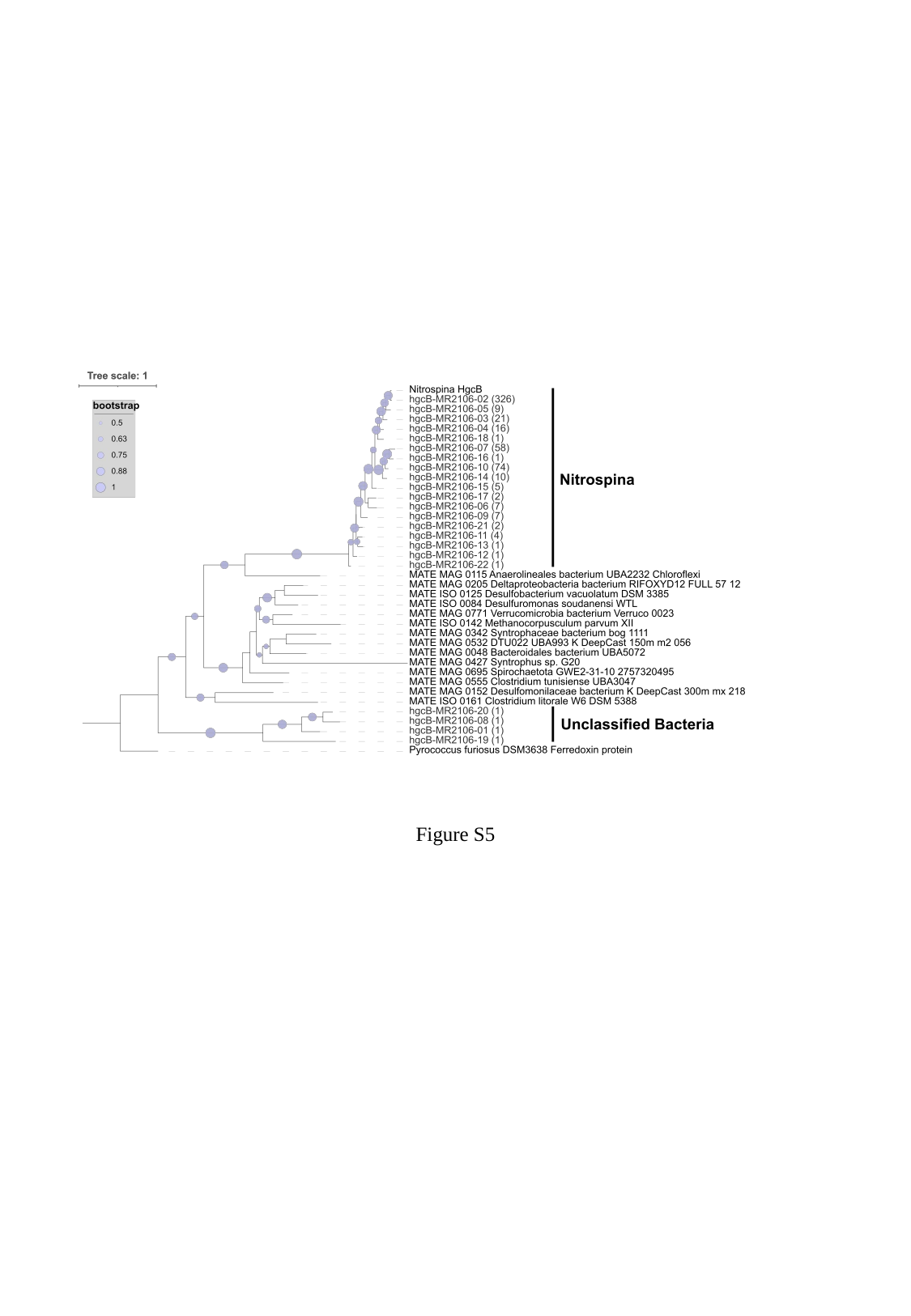

Figure S5

## Slide 6
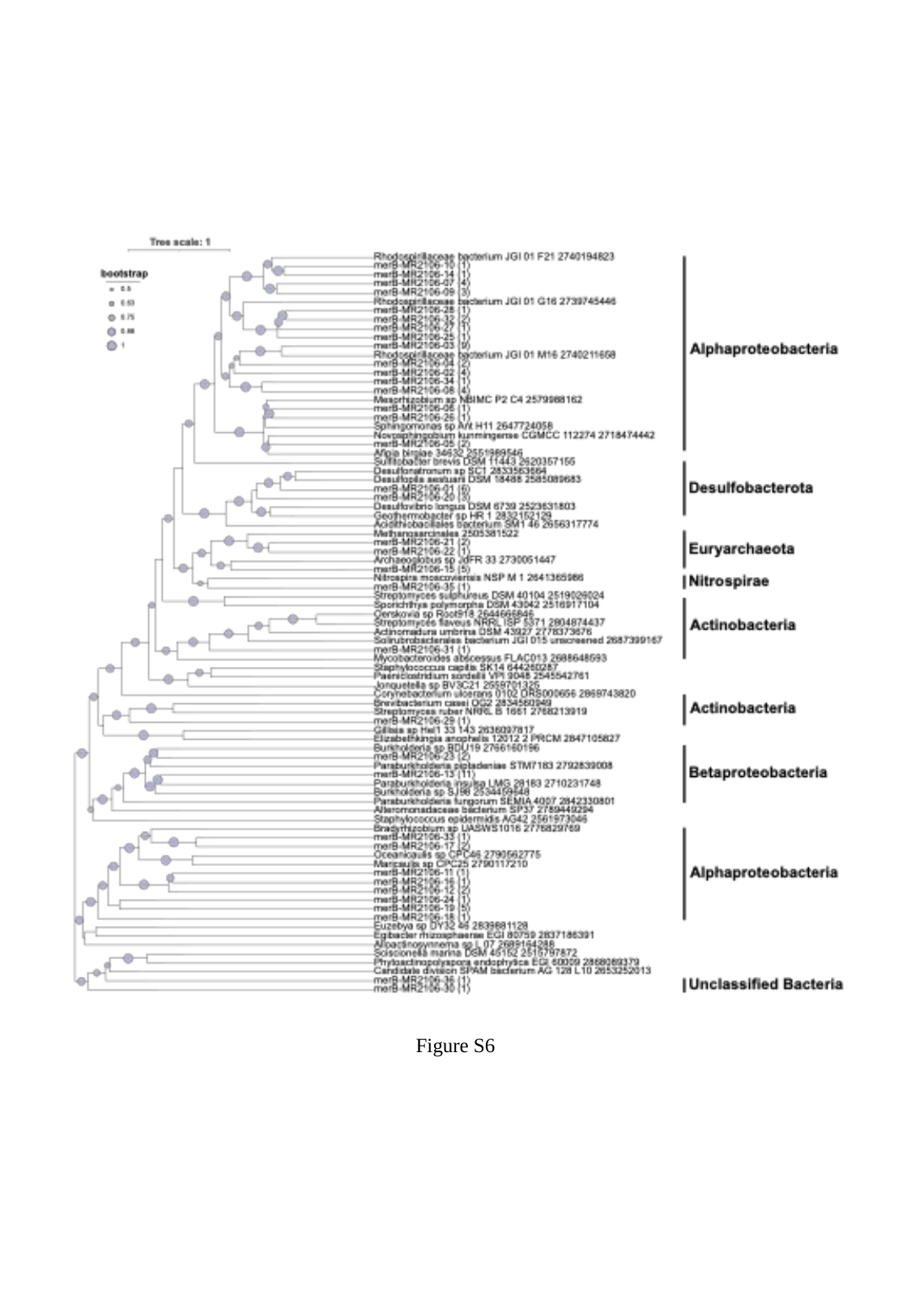

Figure S6

## Slide 7
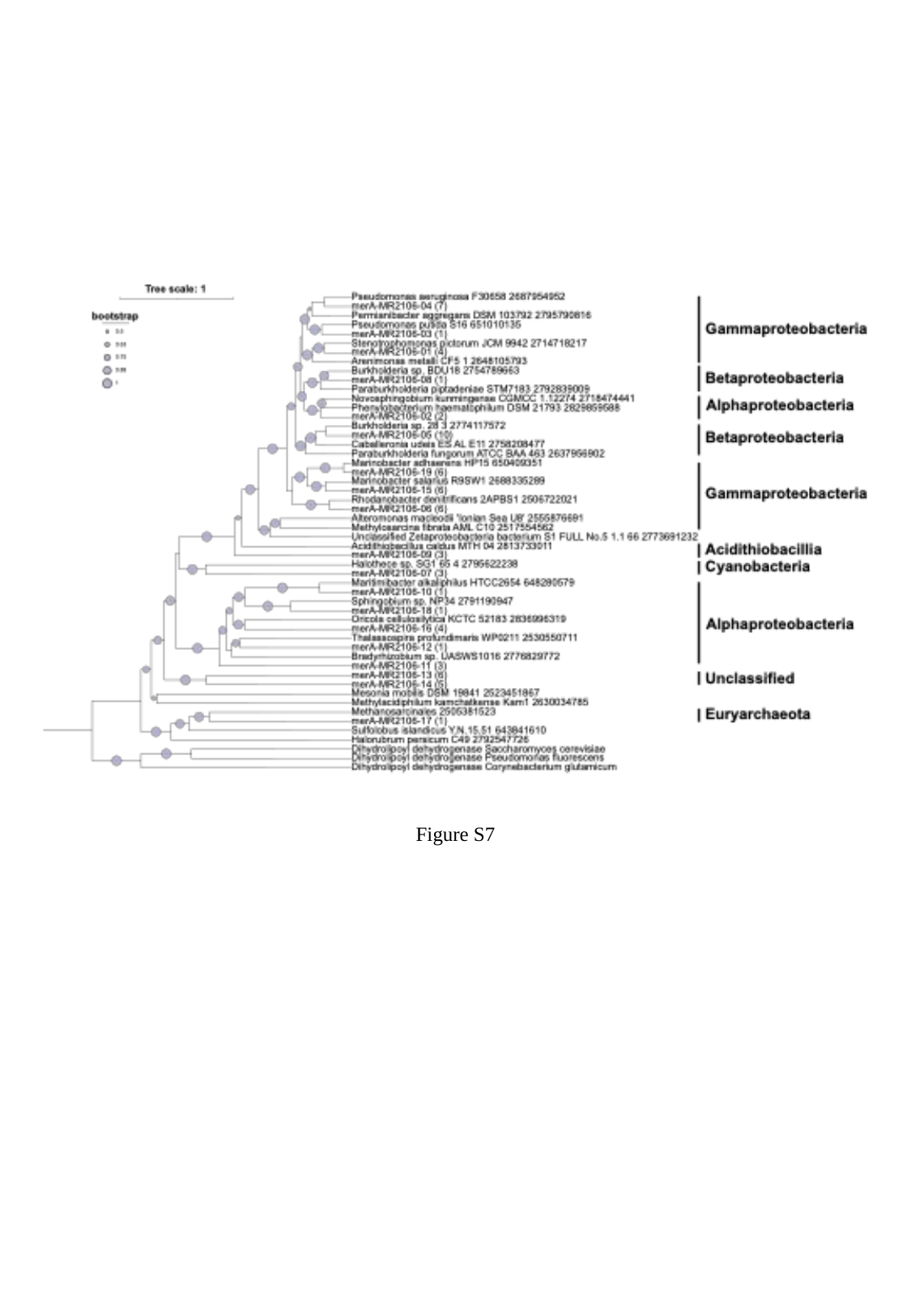

Figure S7

## Slide 8
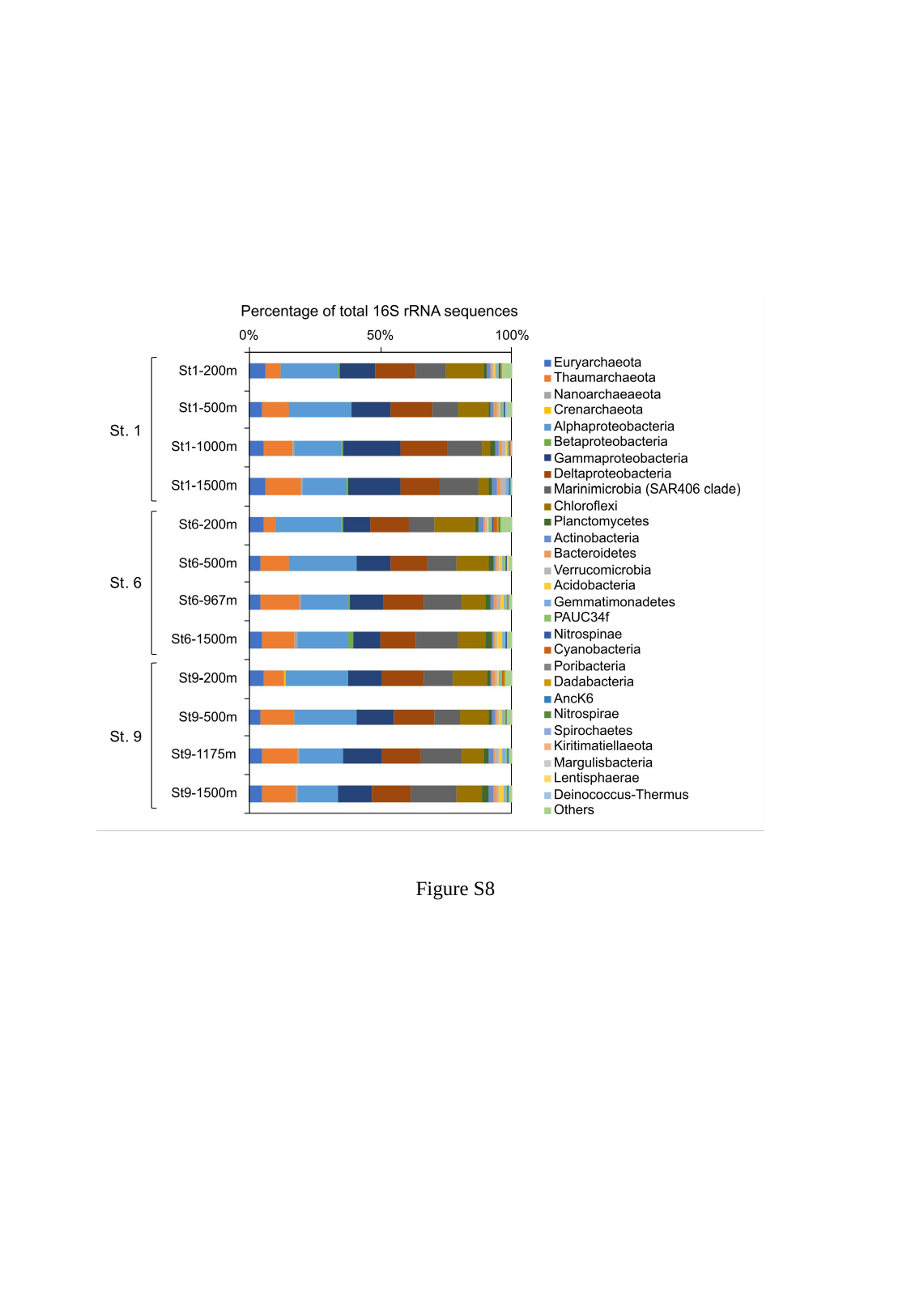

Figure S8

## Slide 9
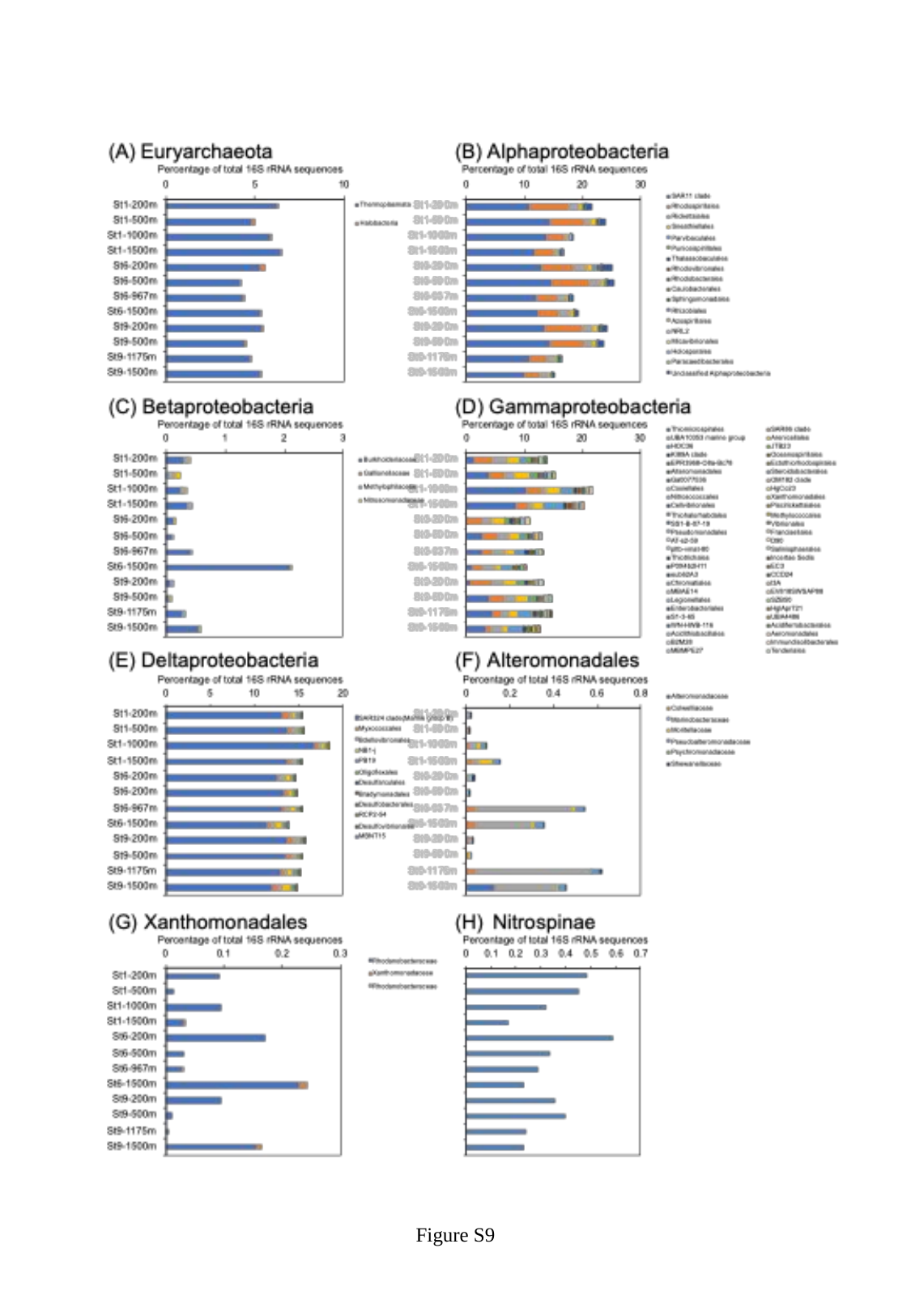

Figure S9
