# Supplementary material for: Distribution and function of prokaryotes involved in mercury methylation, demethylation, and reduction in the western North Pacific Subtropical Gyre
Source: Front Microbiol. 2026 Jan 22;16:1642479. doi: 10.3389/fmicb.2025.1642479 (PMC12874090; doi:10.3389/fmicb.2025.1642479)
Supplement: Supplementary file 11 [file Supplementary_file_1.docx]

Supplementary Material

# Supplementary Figures and Tables

**Table S1.** Information on the metagenomic sequences, contigs, predicted genes, and Hg-related genes in the western North Pacific Subtropical Gyre

| Metagenomic samples | St. 1 |  |  |  |  | St. 6 |  |  |  |  | St. 9 |  |  |  |
| --- | --- | --- | --- | --- | --- | --- | --- | --- | --- | --- | --- | --- | --- | --- |
|  | 200 m | 500 m | 1000 m | 1500 m |  | 200 m | 500 m | 967 m | 1500 m |  | 200 m | 500 m | 1175 m | 1500 m |
| Quantity of metagenomic DNA (ng) | 564 | 632 | 478 | 300 |  | 223 | 160 | 116 | 109 |  | 307 | 470 | 137 | 131 |
| Total read bases (bp) | 109,086,239,024 | 126,506,540,358 | 120,782,123,412 | 125,525,660,062 |  | 105,206,898,326 | 108,455,173,784 | 135,652,290,842 | 102,876,740,014 |  | 127,961,562,466 | 101,316,974,340 | 104,130,411,776 | 102,569,430,250 |
| No. of contigs after MEGAHIT assmbly** | 17,072,250 | 24,100,184 | 19,318,638 | 17,004,876 |  | 14,102,736 | 21,496,452 | 20,175,420 | 13,353,175 |  | 19,830,645 | 18,274,386 | 14,921,079 | 12,867,614 |
| N50 of contigs (bp) | 655 | 547 | 493 | 524 |  | 724 | 563 | 570 | 621 |  | 645 | 563 | 574 | 603 |
| Minimum length of contigs (bp) | 200 | 200 | 200 | 200 |  | 200 | 200 | 200 | 200 |  | 200 | 200 | 200 | 200 |
| Maximum length of contigs (bp) | 923,138 | 1,307,847 | 319,509 | 489,399 |  | 1,028,023 | 750,675 | 725,742 | 834,344 |  | 945,312 | 884,557 | 725,742 | 562,284 |
| Average length of contigs (bp) | 578.9 | 518 | 483.5 | 500.7 |  | 613.5 | 534.1 | 529.6 | 560.5 |  | 569.4 | 532.7 | 534.8 | 552.1 |
| No. of predicted genes | 22,128,090 | 30,359,192 | 23,712,016 | 21,059,212 |  | 18,769,453 | 27,303,771 | 25,448,338 | 17,051,219 |  | 25,712,913 | 23,110,371 | 18,854,888 | 15,470,080 |
| No. of *hgcA* sequences | 1 | 6 | 34 | 33 |  | 1 | 9 | 30 | 14 |  | 1 | 4 | 35 | 34 |
| No. of *hgcB* sequences | 40 | 86 | 57 | 44 |  | 45 | 47 | 39 | 20 |  | 41 | 64 | 36 | 30 |
| No. of *merA* sequences (with Cys207, Cys212, Tyr264, Tyr605 or Phe605, Cys628, and Cys629) | 3 | 1 | 8 | 9 |  | 7 | 1 | 8 | 9 |  | 2 | 0 | 9 | 14 |
| No. of *merB* sequences (with Cys96, Asp99, Cys117, and Cys159) | 3 | 4 | 7 | 9 |  | 3 | 7 | 15 | 12 |  | 4 | 1 | 7 | 15 |
| No. of *rpoB* sequences | 24,930 | 37,734 | 34,306 | 32,454 |  | 21,671 | 22,737 | 18,089 | 10,696 |  | 31,257 | 29,575 | 18,817 | 13,629 |
| No. of MAGs | 115 | 103 | 61 | 83 |  | 136 | 157 | 114 | 114 |  | 136 | 96 | 99 | 94 |
| *Removed the low-quality sequences after paired-end assembly | | | | | | | | | | | |  |  |  |
| **Assembled condition (k-min = 21, k-max = 141, and k-step = 12) | | | | | | | | | | | |  |  |  |

**Table S2.** Phylogenetic lineages of mercury (Hg)-related genes detected from metagenome sequences

| Hg-related gene | Name of sequence | Closest lineage | St1 |  |  |  |  | St6 |  |  |  |  | St9 |  |  |  |
| --- | --- | --- | --- | --- | --- | --- | --- | --- | --- | --- | --- | --- | --- | --- | --- | --- |
|  |  |  | 200 m | 500 m | 1000 m | 1500 m |  | 200 m | 500 m | 967 m | 1500 m |  | 200 m | 500 m | 1175 m | 1500 m |
| *hgcA* | hgcA-MR2106-01 | Deltaproteobacteria bacterium NP36 |  |  | 1 | 3 |  |  |  | 2 |  |  |  |  | 2 | 1 |
|  | hgcA-MR2106-02 | Deltaproteobacteria bacterium NP36 |  |  | 1 | 1 |  |  |  | 1 |  |  |  |  |  |  |
|  | hgcA-MR2106-03 | Deltaproteobacteria bacterium ARS66 |  |  |  |  |  |  |  |  |  |  |  |  | 1 | 1 |
|  | hgcA-MR2106-04 | Deltaproteobacteria bacterium ARS66 |  |  |  | 1 |  |  | 1 | 2 |  |  |  |  | 2 | 2 |
|  | hgcA-MR2106-05 | Deltaproteobacteria bacterium NP36 |  |  | 1 | 1 |  |  | 1 |  |  |  |  |  | 1 | 1 |
|  | hgcA-MR2106-06 | Deltaproteobacteria bacterium SP3084 |  |  | 2 | 1 |  |  |  | 1 |  |  |  |  | 1 |  |
|  | hgcA-MR2106-07 | Deltaproteobacteria bacterium SP3084 |  |  |  | 1 |  |  |  |  |  |  |  |  | 1 | 1 |
|  | hgcA-MR2106-08 | Deltaproteobacteria bacterium SP3084 |  |  |  | 1 |  |  |  |  | 1 |  |  |  | 1 | 1 |
|  | hgcA-MR2106-09 | Deltaproteobacteria bacterium SP3084 |  |  |  | 1 |  |  |  | 4 | 1 |  |  |  | 2 | 2 |
|  | hgcA-MR2106-10 | Deltaproteobacteria bacterium NP36 |  |  |  |  |  |  |  |  | 1 |  |  |  |  |  |
|  | hgcA-MR2106-11 | Nitrospina SCGC AAA288-L16 | 1 | 5 | 21 | 10 |  |  | 5 | 15 | 6 |  | 1 | 2 | 14 | 15 |
|  | hgcA-MR2106-12 | Nitrospina SCGC AAA288-L16 |  | 1 | 1 | 4 |  |  | 1 | 1 |  |  |  | 1 | 2 | 1 |
|  | hgcA-MR2106-13 | Nitrospina SCGC AAA288-L16 |  |  | 6 | 5 |  |  | 1 | 3 | 3 |  |  | 1 | 5 | 4 |
|  | hgcA-MR2106-14 | Nitrospina SCGC AAA288-L16 |  |  | 1 | 1 |  |  |  |  | 1 |  |  |  | 1 | 1 |
|  | hgcA-MR2106-15 | Nitrospina SCGC AAA288-L16 |  |  |  | 1 |  |  |  |  |  |  |  |  |  |  |
|  | hgcA-MR2106-16 | Nitrospina SCGC AAA288-L16 |  |  |  |  |  |  |  | 1 |  |  |  |  |  |  |
|  | hgcA-MR2106-17 | Defluviimonas indica strain DSM 24802 |  |  |  |  |  |  |  | 1 |  |  |  |  |  |  |
|  | hgcA-MR2106-18 | Deltaproteobacteria bacterium NP36 |  |  |  | 1 |  |  |  |  |  |  |  |  |  |  |
|  | hgcA-MR2106-19 | Deltaproteobacteria bacterium NP36 |  |  |  |  |  |  |  |  |  |  |  |  |  | 1 |
|  | hgcA-MR2106-20 | Deltaproteobacteria bacterium NP36 |  |  |  | 1 |  |  |  |  |  |  |  |  |  | 1 |
|  | hgcA-MR2106-21 | Nitrospina SCGC AAA288-L16 |  |  |  |  |  | 1 |  |  |  |  |  |  |  |  |
|  | hgcA-MR2106-22 | Deltaproteobacteria bacterium SP3084 |  |  |  |  |  |  |  |  |  |  |  |  |  | 1 |
|  | hgcA-MR2106-23 | Deltaproteobacteria bacterium SP3084 |  |  |  |  |  | 1 |  |  |  |  |  |  |  |  |
|  | hgcA-MR2106-24 | Nitrospina SCGC AAA288-L16 |  |  |  |  |  |  |  |  |  |  |  |  |  | 1 |
|  | hgcA-MR2106-25 | Deltaproteobacteria bacterium NP36 |  |  |  |  |  |  |  |  |  |  |  |  | 1 |  |
| *hgcB* | hgcB-MR2106-01 | Unclassified |  | 1 |  |  |  |  |  |  |  |  |  |  |  |  |
|  | hgcB-MR2106-02 | Nitrospinae bacterium | 1 | 47 | 51 | 38 |  |  | 37 | 30 | 18 |  | 2 | 48 | 29 | 25 |
|  | hgcB-MR2106-03 | Nitrospinae bacterium | 2 | 4 | 1 | 1 |  | 2 | 3 | 2 |  |  | 2 | 1 | 1 | 2 |
|  | hgcB-MR2106-04 | Nitrospinae bacterium |  | 3 | 1 |  |  |  | 3 | 3 |  |  |  | 4 | 2 |  |
|  | hgcB-MR2106-05 | Nitrospinae bacterium |  | 3 | 3 |  |  | 3 |  |  |  |  |  |  |  |  |
|  | hgcB-MR2106-06 | Nitrospinae bacterium | 2 | 1 |  |  |  |  | 1 |  |  |  | 3 |  |  |  |
|  | hgcB-MR2106-07 | Nitrospinae bacterium | 8 | 5 | 3 | 3 |  | 15 | 1 | 3 |  |  | 12 | 3 | 2 | 3 |
|  | hgcB-MR2106-08 | Unclassified |  |  |  |  |  |  |  |  |  |  |  |  | 1 |  |
|  | hgcB-MR2106-09 | Nitrospinae bacterium | 2 |  |  |  |  | 3 |  |  |  |  | 2 |  |  |  |
|  | hgcB-MR2106-10 | Nitrospinae bacterium | 18 | 16 |  |  |  | 18 | 1 |  |  |  | 16 | 5 |  |  |
|  | hgcB-MR2106-11 | Nitrospinae bacterium | 3 |  |  |  |  |  | 1 |  |  |  |  |  |  |  |
|  | hgcB-MR2106-12 | Nitrospinae bacterium |  |  |  |  |  | 1 |  |  |  |  |  |  |  |  |
|  | hgcB-MR2106-13 | Nitrospinae bacterium |  |  |  |  |  |  |  |  |  |  | 1 |  |  |  |
|  | hgcB-MR2106-14 | Nitrospinae bacterium | 1 | 4 |  |  |  | 1 |  |  |  |  | 2 | 2 |  |  |
|  | hgcB-MR2106-15 | Nitrospinae bacterium |  |  | 1 | 1 |  |  |  | 1 | 1 |  |  |  | 1 |  |
|  | hgcB-MR2106-16 | Nitrospinae bacterium |  |  |  | 1 |  |  |  |  |  |  |  |  |  |  |
|  | hgcB-MR2106-17 | Nitrospinae bacterium |  |  |  |  |  | 1 |  |  |  |  |  | 1 |  |  |
|  | hgcB-MR2106-18 | Nitrospinae bacterium |  |  |  |  |  |  |  |  |  |  |  |  |  | 1 |
|  | hgcB-MR2106-19 | Unclassified |  |  |  |  |  |  |  |  | 1 |  |  |  |  |  |
|  | hgcB-MR2106-20 | Unclassified |  |  |  |  |  | 1 |  |  |  |  |  |  |  |  |
|  | hgcB-MR2106-21 | Nitrospinae bacterium |  | 1 |  |  |  |  |  |  |  |  | 1 |  |  |  |
|  | hgcB-MR2106-22 | Nitrospinae bacterium |  | 1 |  |  |  |  |  |  |  |  |  |  |  |  |
| *merB* | merB-MR2106-01 | Geothermobacter sp HR 1 | 1 | 1 |  |  |  | 1 | 1 |  |  |  | 1 | 1 |  |  |
|  | merB-MR2106-02 | Rhodospirillaceae bacterium JGI 01 M16 |  |  |  | 1 |  |  |  | 1 | 1 |  |  |  |  | 1 |
|  | merB-MR2106-03 | Rhodospirillaceae bacterium JGI 01 M16 |  |  | 1 | 1 |  |  |  | 3 | 1 |  |  |  | 2 | 1 |
|  | merB-MR2106-04 | Rhodospirillaceae bacterium JGI 01 M16 |  |  |  |  |  |  |  |  | 1 |  |  |  |  | 1 |
|  | merB-MR2106-05 | Afipia birgiae 34632 |  |  |  |  |  |  |  | 1 |  |  |  |  | 1 |  |
|  | merB-MR2106-06 | Mesorhizobium sp NBIMC P2 C4 |  |  |  |  |  |  |  |  |  |  |  |  |  | 1 |
|  | merB-MR2106-07 | Rhodospirillaceae bacterium JGI 01 F21 |  |  | 1 |  |  |  |  | 1 |  |  |  |  | 1 | 1 |
|  | merB-MR2106-08 | Rhodospirillaceae bacterium JGI 01 M16 |  |  |  | 1 |  |  |  | 1 | 1 |  |  |  |  | 1 |
|  | merB-MR2106-09 | Rhodospirillaceae bacterium JGI 01 F21 |  |  | 1 |  |  |  | 1 | 1 |  |  |  |  |  |  |
|  | merB-MR2106-10 | Rhodospirillaceae bacterium JGI 01 F21 |  |  |  | 1 |  |  |  |  |  |  |  |  |  |  |
|  | merB-MR2106-11 | Maricaulis sp CPC25 |  |  |  |  |  |  |  |  | 1 |  |  |  |  |  |
|  | merB-MR2106-12 | Maricaulis sp CPC25 |  |  |  | 1 |  |  |  |  |  |  |  |  |  | 1 |
|  | merB-MR2106-13 | Paraburkholderia insulsa LMG 28183 | 1 | 1 | 1 | 1 |  | 1 | 1 | 1 | 1 |  | 1 |  | 1 | 1 |
|  | merB-MR2106-14 | Rhodospirillaceae bacterium JGI 01 F21 |  |  |  |  |  |  |  |  |  |  |  |  |  | 1 |
|  | merB-MR2106-15 | Archaeoglobus sp JdFR 33 |  |  |  |  |  |  | 1 | 1 | 1 |  |  |  | 1 | 1 |
|  | merB-MR2106-16 | Maricaulis sp CPC25 |  |  |  |  |  |  |  | 1 |  |  |  |  |  |  |
|  | merB-MR2106-17 | Bradyrhizobium sp UASWS1016 |  |  |  | 1 |  |  |  | 1 |  |  |  |  |  |  |
|  | merB-MR2106-18 | Maricaulis sp CPC25 |  |  |  |  |  |  |  | 1 |  |  |  |  |  |  |
|  | merB-MR2106-19 | Maricaulis sp CPC25 |  | 1 |  |  |  |  | 1 | 1 |  |  |  |  | 1 | 1 |
|  | merB-MR2106-20 | Desulfopila aestuarii DSM 18488 | 1 | 1 |  |  |  |  |  |  |  |  | 1 |  |  |  |
|  | merB-MR2106-21 | Methanosarcinales |  |  |  |  |  |  |  | 1 | 1 |  |  |  |  |  |
|  | merB-MR2106-22 | Methanosarcinales |  |  |  |  |  |  | 1 |  |  |  |  |  |  |  |
|  | merB-MR2106-23 | Burkholderia sp BDU19 |  |  |  |  |  | 1 |  |  |  |  | 1 |  |  |  |
|  | merB-MR2106-24 | Maricaulis sp CPC25 |  |  | 1 |  |  |  |  |  |  |  |  |  |  |  |
|  | merB-MR2106-25 | Rhodospirillaceae bacterium JGI 01 G16 |  |  |  |  |  |  |  |  | 1 |  |  |  |  |  |
|  | merB-MR2106-26 | Mesorhizobium sp NBIMC P2 C4 |  |  |  |  |  |  |  |  | 1 |  |  |  |  |  |
|  | merB-MR2106-27 | Rhodospirillaceae bacterium JGI 01 G16 |  |  |  |  |  |  |  |  |  |  |  |  |  | 1 |
|  | merB-MR2106-28 | Rhodospirillaceae bacterium JGI 01 G16 |  |  | 1 |  |  |  |  |  |  |  |  |  |  |  |
|  | merB-MR2106-29 | Streptomyces ruber NRRL B 1661 |  |  | 1 |  |  |  |  |  |  |  |  |  |  |  |
|  | merB-MR2106-30 | Unclassified |  |  |  |  |  |  |  |  |  |  |  |  |  | 1 |
|  | merB-MR2106-31 | Solirubrobacterales bacterium JGI 015 |  |  |  |  |  |  | 1 |  |  |  |  |  |  |  |
|  | merB-MR2106-32 | Rhodospirillaceae bacterium JGI 01 G16 |  |  |  |  |  |  |  |  | 1 |  |  |  |  | 1 |
|  | merB-MR2106-33 | Bradyrhizobium sp UASWS1016 |  |  |  |  |  |  |  |  | 1 |  |  |  |  |  |
|  | merB-MR2106-34 | Rhodospirillaceae bacterium JGI 01 M16 |  |  |  | 1 |  |  |  |  |  |  |  |  |  |  |
|  | merB-MR2106-35 | Nitrospira moscoviensis NSP M 1 |  |  |  | 1 |  |  |  |  |  |  |  |  |  |  |
|  | merB-MR2106-36 | Unclassified |  |  |  |  |  |  |  |  |  |  |  |  |  | 1 |
| *merA* | merA-MR2106-01 | Stenotrophomonas pictorum JCM 9942 |  |  |  |  |  | 1 |  |  | 1 |  | 1 |  |  | 1 |
|  | merA-MR2106-02 | Phenylobacterium haematophilum DSM 21793 |  |  |  |  |  |  |  | 1 |  |  |  |  | 1 |  |
|  | merA-MR2106-03 | Pseudomonas putida S16 |  |  |  |  |  |  |  |  | 1 |  |  |  |  |  |
|  | merA-MR2106-04 | Pseudomonas aeruginosa F30658 | 1 |  | 1 | 1 |  | 1 |  | 1 | 1 |  |  |  |  | 1 |
|  | merA-MR2106-05 | Burkholderia sp. 28 3 | 1 | 1 | 1 | 1 |  | 1 | 1 | 1 | 1 |  |  |  | 1 | 1 |
|  | merA-MR2106-06 | Rhodanobacter denitrificans 2APBS1 | 1 |  | 1 | 1 |  | 1 |  |  |  |  | 1 |  |  | 1 |
|  | merA-MR2106-07 | Halothece sp. SG1 65 4 |  |  |  |  |  |  |  |  | 1 |  |  |  | 1 | 1 |
|  | merA-MR2106-08 | Burkholderia sp. BDU18 |  |  |  |  |  | 1 |  |  |  |  |  |  |  |  |
|  | merA-MR2106-09 | Acidithiobacillus caldus MTH 04 |  |  |  |  |  |  |  | 1 |  |  |  |  | 1 | 1 |
|  | merA-MR2106-10 | Maritimibacter alkaliphilus HTCC2654 |  |  |  |  |  |  |  |  |  |  |  |  |  | 1 |
|  | merA-MR2106-11 | Bradyrhizobium sp. UASWS1016 |  |  |  | 1 |  |  |  |  | 1 |  |  |  |  | 1 |
|  | merA-MR2106-12 | Thalassospira profundimaris WP0211 |  |  |  |  |  |  |  |  | 1 |  |  |  |  |  |
|  | merA-MR2106-13 | Unclassified |  |  | 1 | 1 |  |  |  | 1 | 1 |  |  |  | 1 | 1 |
|  | merA-MR2106-14 | Unclassified |  |  | 1 | 1 |  |  |  | 1 | 1 |  |  |  |  | 1 |
|  | merA-MR2106-15 | Marinobacter salarius R9SW1 |  |  | 1 | 1 |  | 1 |  |  | 1 |  |  |  | 1 | 1 |
|  | merA-MR2106-16 | Oricola cellulosilytica KCTC 52183 |  |  | 1 | 1 |  |  |  |  |  |  |  |  | 1 | 1 |
|  | merA-MR2106-17 | Methanosarcinales |  |  |  |  |  |  |  |  |  |  |  |  |  | 1 |
|  | merA-MR2106-18 | Sphingobium sp. NP34 |  |  |  |  |  |  |  | 1 |  |  |  |  |  |  |
|  | merA-MR2106-19 | Marinobacter adhaerens HP15 |  |  | 1 | 1 |  | 1 |  | 1 |  |  |  |  | 1 | 1 |
|  |  |  |  |  |  |  |  |  |  |  |  |  |  |  |  |  |

**Figure S1.** Map of sampling stations in the western North Pacific Subtropical Gyre

**Figure S2.** Alignment of conserved regions in amino acid sequences of merA (**A**) and merB (**B**) genes detected in this study.

**Figure S3.** Relationship between the proportion of *hgcA* and *merB* (**A**), *hgcA* and *merA* (**B**), and *merA* and *merB* (**C**)

**Figure S4.** Maximum likelihood phylogenetic tree of the *hgcA* sequences detected in this study. Identified sequences were compared with *hgcA* homologs described in a previous study (Gionfriddo et al., 2019). The *hgcA* paralogs from non-methylators are used for the tree root. Bootstrap values > 50 are represented, with consensus based on 100 replicates.

**Figure S5.** Maximum likelihood phylogenetic tree of the *hgcB* sequences detected in this study. Identified sequences were compared with *hgcB* homologs described in a previous study (Gionfriddo et al., 2019). Bootstrap values > 50 are represented, with consensus based on 100 replicates.

**Figure S6.** Maximum likelihood phylogenetic tree of the *merB* sequences detected in this study. Identified sequences were compared with *hgcB* homologs described in a previous study (Christakis et al., 2021). Bootstrap values > 50 are represented, with consensus based on 100 replicates.

**Figure S7.** Maximum likelihood phylogenetic tree of the *merA* sequences detected in this study. Identified sequences were compared with *merA* homologs described in a previous study (Christakis et al., 2021). The dihydrolipoyl dehydrogenase sequences (presumably *merA* paralogs) are used for the tree root. Bootstrap values > 50 are represented, with consensus based on 100 replicates.

**Figure S8.** Prokaryotic community structure based on 16S rRNA gene analysis at each station and depth

**Figure S9.** Details of the community structure based on 16S rRNA gene analysis at each station and depth
